# Interactions between Hydrolysable Tannins and Lipid Vesicles from *Escherichia coli* with Isothermal Titration Calorimetry

**DOI:** 10.3390/molecules27103204

**Published:** 2022-05-17

**Authors:** Valtteri Virtanen, Rebecca J. Green, Maarit Karonen

**Affiliations:** 1 Natural Chemistry Research Group, Department of Chemistry, University of Turku, FI-20014 Turku, Finland; maarit.karonen@utu.fi; 2 School of Chemistry, Food and Pharmacy, University of Reading, Whiteknights, P.O. Box 224, Reading RG6 6AP, UK; rebecca.green@reading.ac.uk

**Keywords:** ellagitannin, *E. coli*, gallotannin, isothermal titration calorimetry, lipid, lipid bilayer, thermodynamics, polyphenol

## Abstract

Isothermal titration calorimetry (ITC) was used to study the interactions between hydrolysable tannins (HTs) and lipid vesicles prepared from a phospholipid extract of *Escherichia coli* (*E. coli*). A group of 24 structurally different HTs was selected, and structural differences affecting their affinities to interact with lipid vesicles in aqueous buffered media were identified. In general, the interactions between HTs and lipid vesicles were exothermic in nature, and ITC as a technique functioned well in the screening of HTs for their affinity for lipids. Most notably, the galloyl moiety, the structural flexibility of the entire tannin structure, the hydrophobicity of the tannin, and higher molecular weight were observed to be important for the stronger interactions with the lipids. The strongest interactions with lipids were observed for rugosins D and G. It was also observed that some HTs with moderate hydrophobicities, such as geraniin, chebulagic acid, and chebulinic acid, did not have any detectable interactions with the lipid vesicles, suggesting that a hydrophobic structure alone does not guarantee an affinity for lipids.

## 1. Introduction

Plants produce a large number of chemical compounds with various useful properties. One of the most interesting compound groups is hydrolysable tannins (HT). They possess beneficial properties, such as high protein binding capacity, high oxidative activity, and the capacity to act as antibacterial and antiparasitic agents [1,2,3,4,5,6,7]. The bioactivities of tannins are linked with their interactions with other macromolecules, such as proteins, lipids, and carbohydrates [8,9]. The interactions of HTs with lipids have not been studied nearly to the extent that HT–protein interactions have.

Isothermal titration calorimetry (ITC) is a technique, which enables the study of the thermodynamic properties and interactions of various biological samples. ITC can be utilized to determine the stoichiometry of the reactants and the enthalpy and binding constant of the reaction. ITC has been previously utilized in HT–protein interaction studies and also in studies of various model lipids’ interactions with other lipids, proteins, peptides, metals, and surfactants [1,10,11,12,13,14,15,16,17,18,19,20,21,22].

**Figure 1:**
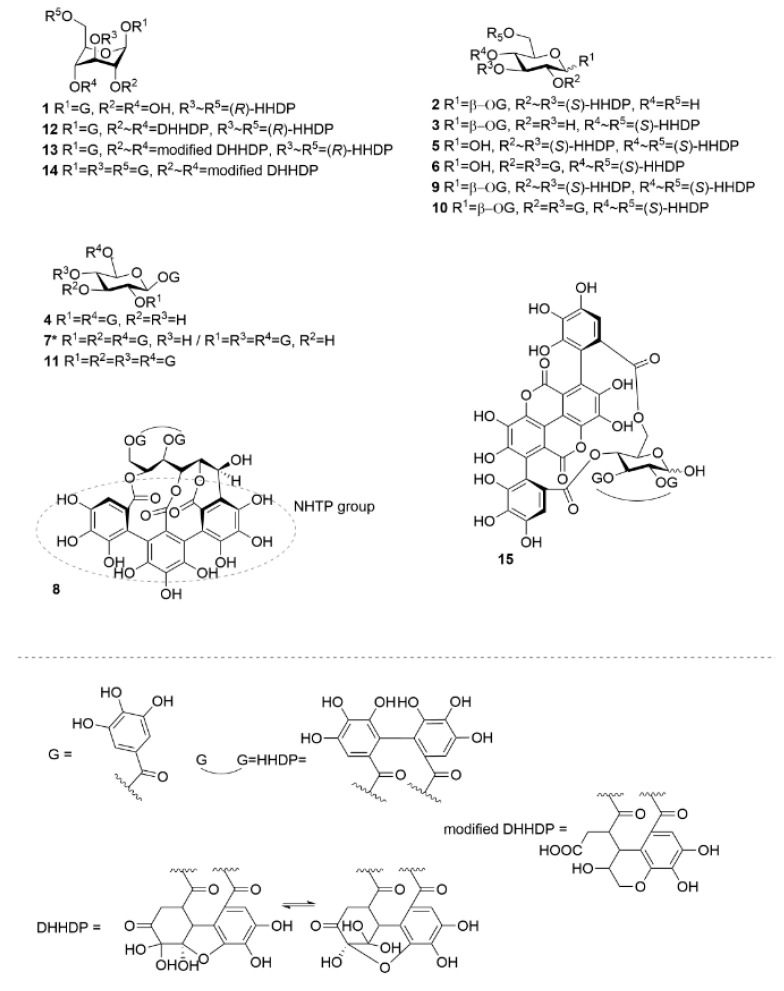
Chemical structures of monomeric hydrolysable tannins used in the study. DHHDP = dehydrohexahydroxydiphenoyl, G = galloyl, HHDP = hexahydroxydiphenoyl, NHTP = nonahydroxytriphenoyl. **1**, corilagin; **2**, isostrictinin; **3**, strictinin; **4**, 1,2,6-tri-*O*-galloyl-β-d-glucose; **5**, pedunculagin; **6**, tellimagrandin I; **7**, 1,2,3,6-tetra-*O*-galloyl-β-d-glucose/1,2,4,6-tetra-*O*-galloyl-β-d-glucose; **8**, vescalagin; **9**, casuarictin; **10**, tellimagrandin II, **11**, 1,2,3,4,6-penta-*O*-galloyl-β-d-glucose; **12**, geraniin, **13**, chebulagic acid; **14**, chebulinic acid; **15**, punicalagin. Compound **7** was used as a mixture (0.42:1.00, n:n) of two structural isomers of tetragalloylglucose.

**Figure 2:**
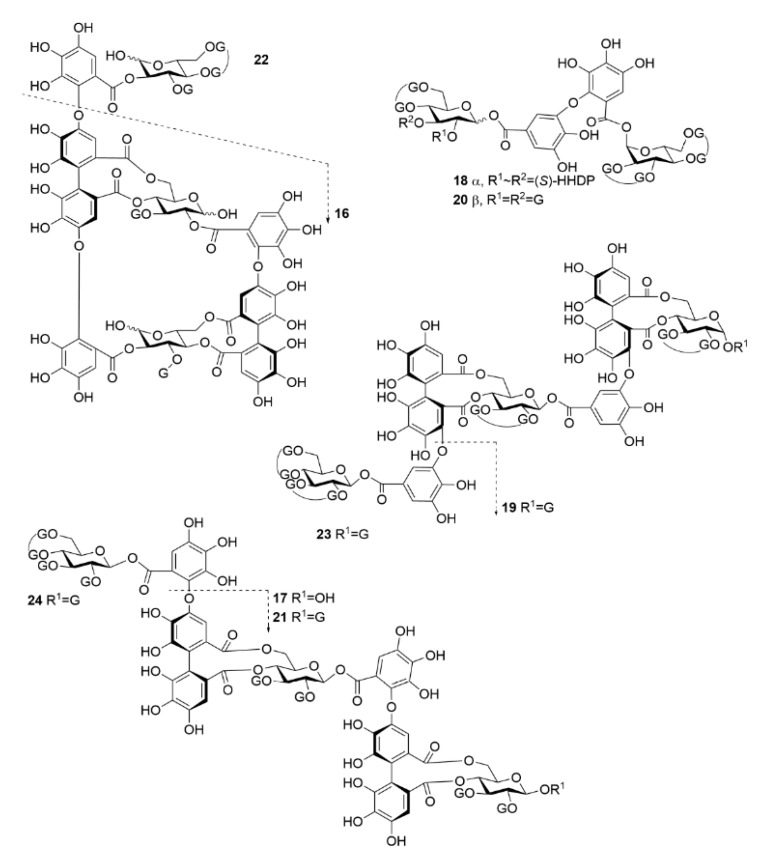
Chemical structures of oligomeric hydrolysable tannins used in the study. **16**, oenothein B; **17**, rugosin E; **18**, agrimoniin; **19**, sanguiin H-6; **20**, gemin A; **21**, rugosin D; **22**, oenothein A; **23**, lambertianin C; **24**, rugosin G. See Figure 1 for the structures of the substitute groups.

In this study, the thermodynamics of the interactions of a select and structurally diverse group of HTs (Figure 1 and Figure 2) with a biomimetic lipid vesicle solution prepared from a lipid extract of *Escherichia coli* (*E. coli*) were investigated. By utilizing ITC, we were able to uncover new information on the interactions of HTs and lipid vesicles and how the structure of the HT can reveal whether it will interact with lipids in buffered aqueous media or if it mainly remains free and non-interactive in solution. To our knowledge, this is the first time the interactions of HTs, or more broadly the interactions of any polyphenol, with lipids have been studied with ITC.

## 2. Results and Discussion

### 2.1. Thermodynamic Screening Method for HT–Lipid Interactions with ITC

**Figure 3:**
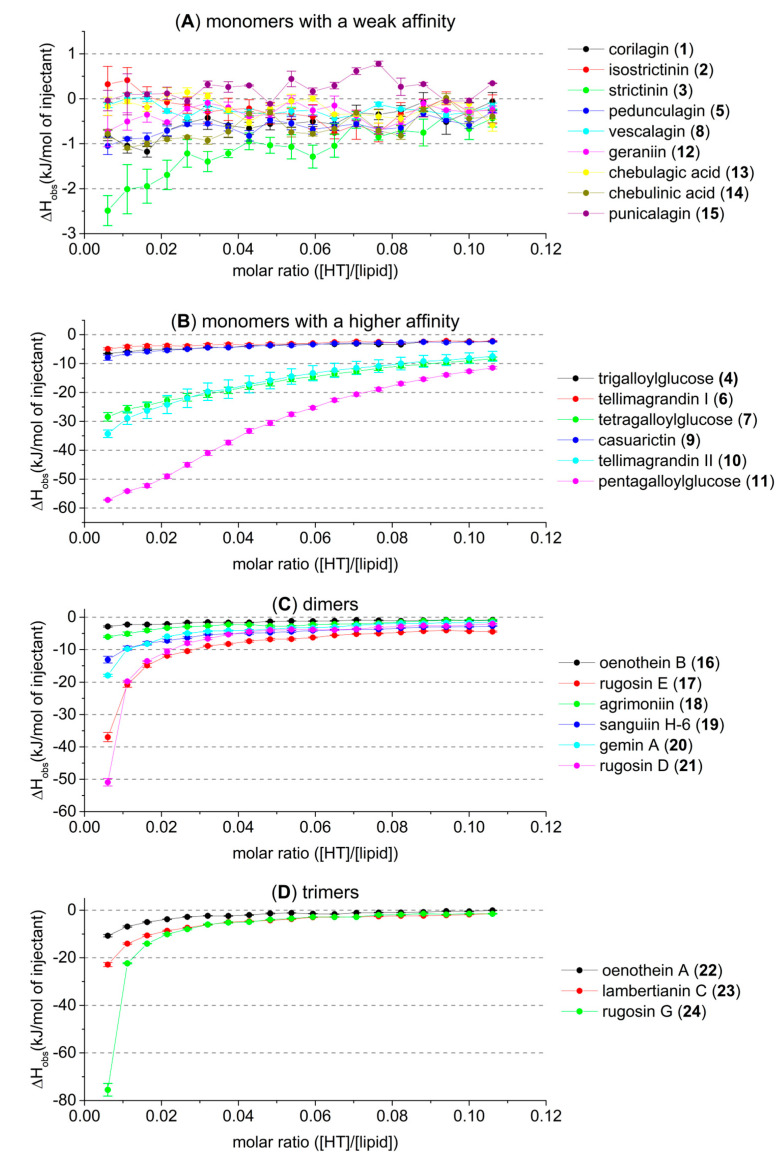
Observed heat rates of injection of studied hydrolysable tannins (HTs) when titrated into a 2 mM *E. coli* lipid vesicle solution as a function of the molar ratio ([HT]/[lipid]) in the sample cell: (**A**) monomers with a weak affinity; (**B**) monomers with a higher affinity; (**C**) dimeric and (**D**) trimeric HTs. Heat rates presented as kJ/mol of injectant with average values and standard error, *n* = 3. For HT structures refer to Figure 1 and Figure 2.

The aim of this study was to further verify and determine which types of structural features of HTs define the tendency of the HTs to interact with lipid vesicles. ITC was found to be a suitable technique for this purpose. In a typical measurement of the interaction between HT and lipids, the lipid solution was placed in the sample cell of the calorimeter, and the HT solution was titrated into the lipid. In HT–lipid interactions, both the structural features and concentrations of HTs and lipids have an effect; therefore, different buffer solutions, different HT concentrations, and different lipid concentrations were tested in order to optimize measurement conditions. Therefore, in order to acquire adequately strong signals, i.e., reasonable heats of injection, which would allow us to find meaningful differences between stronger and weaker interacting HTs, we needed to make compromises on ideal concentrations. It was decided to use concentrations which allowed us to qualitatively study as many different HTs as possible and in so doing define many structural features of HTs that are essential to the HT–lipid interaction. The optimized ITC method used 1 mM HT solutions and a 2 mM lipid solution in 20 mM sodiumphosphate buffer at pH 7. With these concentrations, the heats measured varied from −80 to 0 kJ/mol of injectant; the measurements were reliable and repeatable, and the data informative. The ITC method allowed the qualitative comparison of interactions of different HTs with lipid vesicles (Figure 3), and we could classify them based on the strength of the interaction, as discussed in Section 2.2.

The used concentrations were selected to allow the screening of all different HT structures. However, they did not allow us to perform the quantitative fitting of ITC data reliably, nor to obtain thermodynamic binding parameters. The observed heat rate vs. molar ratio graphs were missing sigmoidal curve shapes (Figure 3), which hindered the fitting process and caused uncertainties in the fitting parameters. Therefore, it was decided it would be better to draw qualitative conclusions on the HT–lipid interactions based on the observed heat rates of injection than to report the estimated binding parameters with uncertainty.

**Figure A1:**
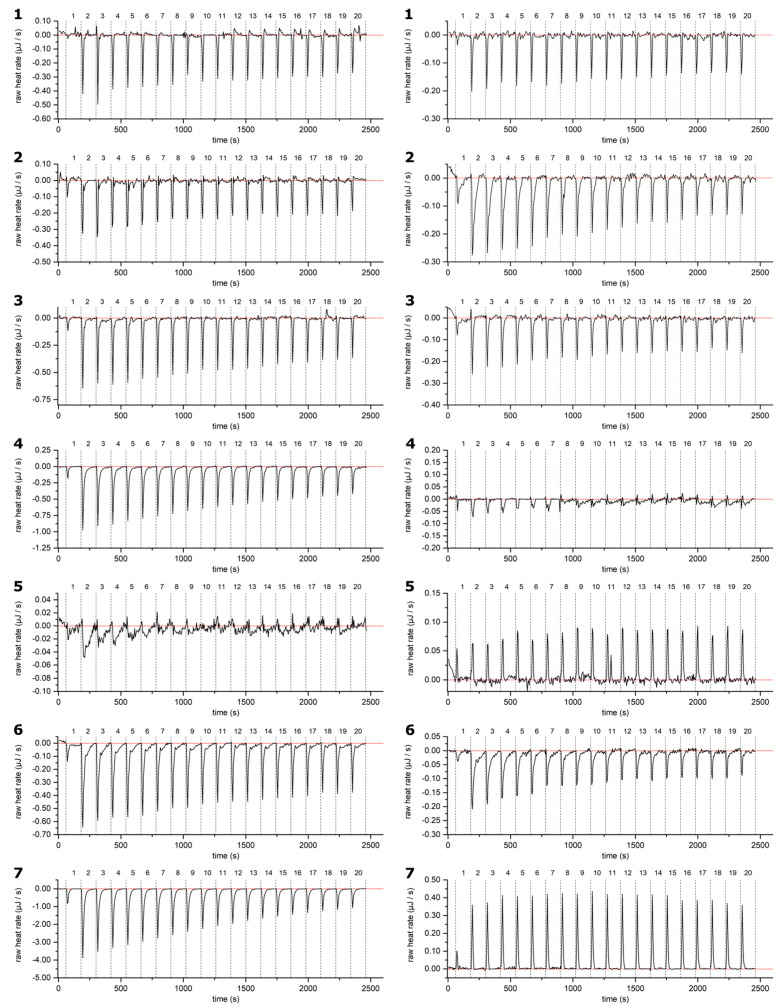
Example thermograms of HTs **1**–**7:** samples (**left**) and control (**right**, HT titrated into buffer solution): measurements plotted against measurement time. Corrected baseline is presented as a red line, and injections are separated with dashed lines. HTs are presented in order of ascending molecular weight. For the structures and numbering of HTs, refer to Figure 1 and Figure 2.

**Figure A2:**
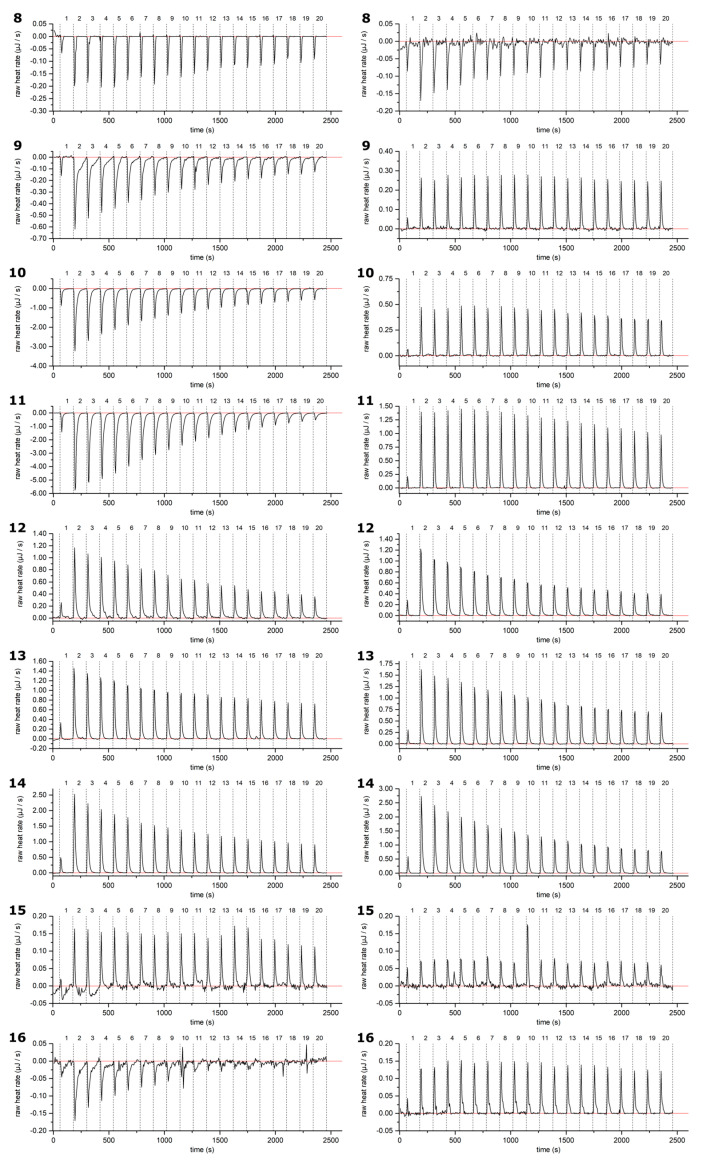
Example thermograms of HTs **8**–**16** sample (**left**) and control (**right**, HT titrated into buffer solution): measurements plotted against measurement time. Corrected baseline presented as a red line, and injections separated with dashed lines. HTs are presented in order of ascending molecular weight. For the structures and numbering of HTs, refer to Figure 1 and Figure 2.

**Figure A3:**
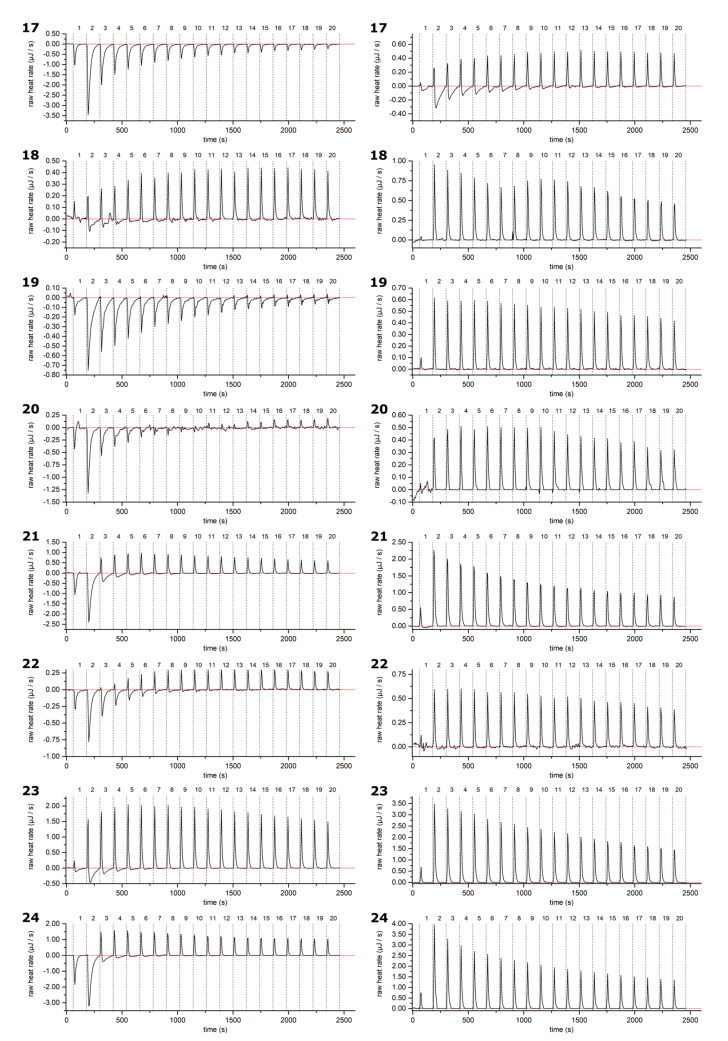
Example thermograms of HTs **17**–**24** samples (**left**) and control (**right**, HT titrated into buffer solution): measurements plotted against measurement time. Corrected baseline presented as a red line, and injections separated with dashed lines. HTs are presented in order of ascending molecular weight. For the structures and numbering of HTs, refer to Figure 1 and Figure 2.

The control measurement of HT titrated into buffer solution (Appendix A Figure A1, Figure A2 and Figure A3, Supplementary Materials Figures S2–S5) varied relatively strongly depending on the studied HT; some produced small exothermic signals, but most produced endothermic signals which were not always constant throughout the injections. HTs are known to self-associate into aggregates in a concentrated HT solution [10,23,24]. The progressively decreasing endothermic heat is attributed to a deaggregation process of HTs when they are titrated into a buffer solution [10,23,24]. The amount of heat from deaggregation will reduce with the injections as the amount of HTs already present in the sample cell increases. Therefore, in the HT–lipid titrations there are two simultaneous processes, i.e., the deaggregation of HTs and their lipid interactions. However, this phenomenon was not a problem, as this deaggregation process could be taken into account by subtracting the control data of each HT titrated into buffer.

### 2.2. HT–Lipid Interaction with ITC

The thermodynamics of the interactions between HTs and lipids were studied with ITC using a set of 22 HTs (Figure 1 and Figure 2). After background subtraction, all measured HTs showed varying amounts of exothermic heat when titrated into the *E. coli* lipid vesicle solution. The idea was that the higher the heat the addition of each HT produces is an indication of the degree to which the HT is able to interact with the lipid vesicles, or inversely if the HT molecules do not interact with lipid vesicles. Similarly, in our recent high-resolution magic angle spinning nuclear magnetic resonance (HR-MAS NMR) spectroscopic study using the same lipid extract and a subset of the same HTs used in this study, the importance of certain structural features was observed [25]. The initial premise was that the same features would prove dominant in this thermodynamic study as well. However, a wider range of HT structures could be studied in this case with ITC, as the measurements were faster and required smaller amounts of HTs than in the previous NMR study. This all facilitated more precise observations of how the HTs’ structures affect their interactions with lipids. In general, it was noticed that the amount of heat per mole of HT injected into the lipid vesicles was in a similar range, up to −80 kJ/mol of injectant (Figure 3), to what has been previously reported for HT–protein, proanthocyanidin–protein, and other polyphenol–protein interactions by ITC [1,10,26,27].

The monomeric HTs could be classified as monomers with weak affinity (Figure 3A) and monomers with high affinity for lipids (Figure 3B). The observed heat rates of the interactions between the most active HT monomers and lipids were roughly in the same level as the interactions between HT dimers and trimers and lipids (Figure 3C,D).

There was a distinct trend in the monomeric HTs (Figure 3B) where if the number of free galloyl groups in the HT structure decreased, for example, when moving from pentagalloylglucose (**11**) → tetragalloylglucose (**7**) → trigalloylglucose (**4**), the amount of detected heat also decreased considerably. The galloyl group(s) in the HT structures clearly increased the interaction with the lipids, which is logical, as the number of galloyl groups also influences the hydrophobicity of HTs [28,29]. The same decreasing effect on the heat can be seen when the free galloyls bind via C–C bonds, forming considerably more rigid HHDP groups in the HT structures. This can be seen in the series of pentagalloylglucose (**11**) → tellimagrandin II (**8**) → casuarictin (**9**) and tetragalloylglucose (**7**) → tellimagrandin I (**6**) → pedunculagin (**5**). The two free galloyl groups instead of one HHDP group makes the interactions between HTs and lipids stronger, which can be due to different reasons, i.e., the presence and nature of the galloyl group itself or the higher flexibility of the HT molecule, or both. In general, the role of galloyl group seems to be an important structural feature affecting the lipid membrane interactions of various polyphenols [30,31]. The more galloyl groups in the structures, the deeper the polyphenol is in the lipid bilayer. As a side note, the series of HTs denoted with arrows is not meant to represent how these compounds are biosynthetically formed in plants but rather as a series of structures to be compared.

Smaller monomeric HTs, corilagin (**1**), isostrictinin (**2**), and strictinin (**3**), produced barely measurable heat with the studied concentration, indicating weak interactions with lipids (Figure 3A). Due to the weak interactions, it was difficult to make reliable structural conclusions solely based on the small differences in activity between HTs **1–3.** However, when these monomeric HTs are compared to a similar sized trigalloylglucose (**4**), which produced a more significant heat, the weaker lipid interactions due to the HHDP groups in compounds **1**–**3** is seen, even in this structure comparison to some extent. Acyclic vescalagin (**8**) produced almost no detectable heat, indicating that there were minor or no interactions with the lipids. This correlated with our previous results. Vescalagin is highly hydrophilic and has not shown any interaction with the lipids studied by HR-MAS NMR measurements [25,28]. The ^1^C_4_ ellagitannins (**12**–**15**) also produced heats that were barely detectable, which was expected based on the results of our previous NMR study, where it was shown that geraniin (**12**) did not move spatially close to these phospholipid vesicles [25]. This result was still unexpected because compounds **12**–**14** are moderately hydrophobic, which suggests that high hydrophobicity does not necessarily dictate if a compound will have high affinity to interact with lipid vesicles [28,29]. This observation also agrees regarding the protein interactions of geraniin (**12**); it has not had as high activity as other similarly sized HTs [1,32]. It is interesting to observe that even though the macromolecules in question (lipid vesicles vs. proteins) are different, there seem to be similar patterns and structural features which indicate how strong their detected interactions are. Additionally, similar patterns have also been detected regarding tannin–polysaccharide interactions, and the importance of galloyl groups was also observed [8].

The larger dimeric (Figure 3C) and trimeric (Figure 3D) HTs also emphasized the role of galloyl groups in the structures, as opposed to HHDP groups, regarding the strength of the interaction, i.e., the amount of heat produced. However, it has to be mentioned that the comparison of the structures of HT oligomers is not as straightforward as with HT monomers, as there are many different structural features to be taken into account. The diversity of HT oligomers derives mainly from the changing number of galloyl or HHDP groups and the conformation of the anomeric position, in addition to the different types of oligomeric linkages. Rugosin D (**21**) produced the most heat and was the most active one out of all the measured dimers, most probably because it has five free galloyl groups in its structure. Following the same logic, the second most effective dimer, rugosin E (**17**), had four free galloyl groups, and these enabled it to interact even more efficiently than the larger dimers, **18–20**. The third most effective dimer, gemin A (**20**), has only two free galloyl groups, and following that, sanguiin H-6 (**19**) and agrimoniin (**18**), have one and none, respectively. From these results, it can be seen that all of the dimers, **17**–**21**, were in order according to how many free galloyl groups there are in their structures; i.e., the HT with a structure with more flexible galloyl groups produces more heat when titrated into lipid vesicle solution. Oenothein B (**16**), however, does not follow this logic because there are two free galloyl groups in its structure, but it is clearly the least effective of all the dimers at interacting with the lipid vesicles. This can be attributed to the rigid macrocyclic structure formed by the two DOG-type oligomeric linkages. It was also noticed that the galloyl groups bound in the oligomeric linkages did not increase the interaction with the lipids as the free galloyl groups did (**18** vs. **19**).

The lipid interactions of three trimeric HTs were measured, and they also followed the same patterns observed for monomeric and dimeric HTs: having more free galloyl groups in the structure predicts higher affinity for lipids. Rugosin G (**24**) produced the most heat out of all the measured HTs, which was expected, considering that it has seven free galloyl groups in its structure. The decrease in the observed heat rates between lambertianin C (**23**) and **24** was large but well reflected by the fact that lambertianin C has only one free galloyl group in its structure. The difference in the measured heats between oenothein A (**22**) and **23** was small, but the more flexible non-macrocyclic structure of **23** enabled it to more effectively interact with the lipid vesicles, even though it has fewer free galloyl groups. This observation again supports the assumption that, in addition to the hydrophobicity caused by the free galloyl groups, the flexibility of the structure is highly important for the penetration of the HTs into lipid bilayers.

**Figure 4:**
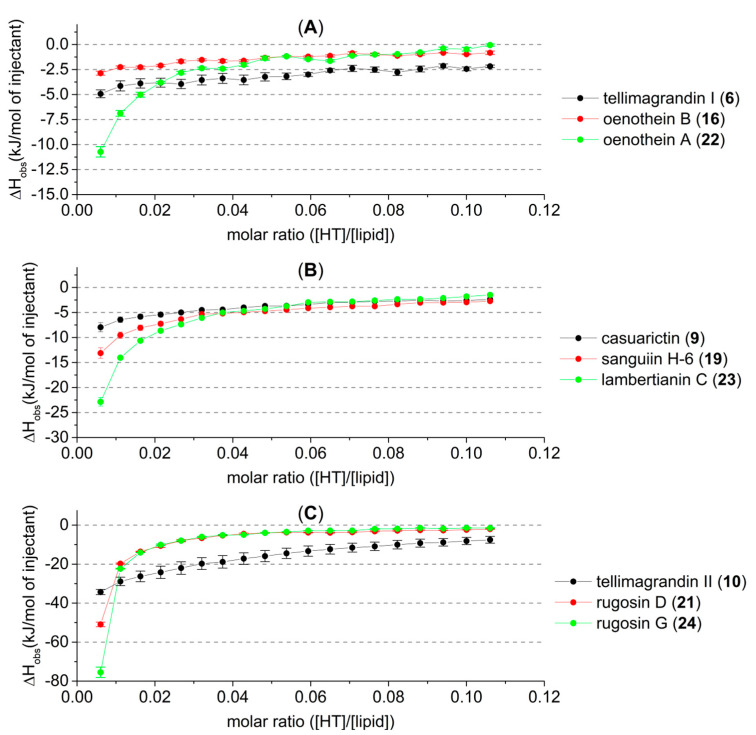
Observed heat rates of injection of three oligomeric series of hydrolysable tannins (HTs) as a function of the molar ratio ([HT]/[lipid]) in the sample cell. Heat rates are presented as kJ/mol of injectant with average values and standard error, *n* = 3. Monomer is presented in black, the dimer in red, and the trimer in green. These oligomeric series are formed from (**A**) tellimagrandin I (**6**), (**B**) casuarictin (**9**), and (**C**) tellimagrandin II (**10**)-type monomeric constituents. For HT structures, refer to Figure 1 and Figure 2.

In the studied HTs, there were three oligomeric series with a varying monomeric unit (Figure 4). By molecular weight, the smallest of these series (**A**) is formed from tellimagandin I (**6**) subunits. As mentioned before, the macrocyclic structure of the dimer oenothein B (**16**) seems to hinder its capability to interact with lipid vesicles. Here it can be seen that even its flexible monomeric unit (**6**) has a higher affinity to lipid vesicles than the rigid dimer. The trimeric oenothein A (**22**) is formed from the dimer so that the additional monomeric unit is attached via only a single DOG type bond; i.e., the added monomeric unit is like a “flexible tail” and can freely rotate, which can increase its affinity above that of its monomeric constituent. The same order of affinity (dimer < monomer < trimer) for this same oligomeric series has also been detected in previous ITC studies on their interactions with proteins [1].

**Figure 5:**
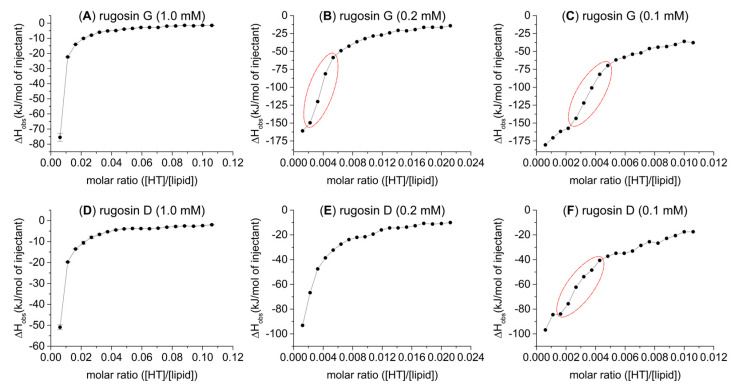
Observed heat rates of injection of 1.0 mM (**A**,**D**), 0.2 mM (**B**,**E**), and 0.1 mM (**C**,**F**) rugosin G (**24**) and rugosin D (**21**) as functions of the molar ratio ([HT]/[lipid]) in the sample cell. Heat rates presented as kJ/mol. Increased sigmoidal curve regions highlighted with red in (**B**,**C**,**F**).

The amounts of heat produced by the second oligomeric series of HTs (**B**) were in the order of increasing oligomerization; the affinity to lipids increased as the molecular size increased. Note here that in the dimer and trimer, one of the terminating monomeric units is the C1 α epimer of casuarictin (**9**); therefore, the series is not entirely homogenous. The dimer and trimer of the third HT series (**C**) were so effective at interacting with the lipid vesicles that, with the concentrations used, their interactions with the lipids almost reached saturation after the first five or six injections. However, these compounds proved to be the most effective structures of their respective oligomer sizes by the observed heat of up to −80 kJ/mol of injectant. It was therefore decided to study their lipid interactions in more depth with lower HT concentrations (Figure 5). By lowering the concentrations of these HTs with very high lipid affinity, we were able to achieve more sigmoid-like thermogram curves, especially with rugosin G (**24**) at 0.2 and 0.1 mM and rugosin D (**21**) at 0.1 mM.

### 2.3. Particle Size Measurements with Dynamic Light Scattering (DLS) 

In the HT–lipid interactions, both the structural features and concentrations of HTs and lipids have an effect. Therefore, in addition to the exact characterization of HTs, we wanted to carefully characterize the used model lipid vesicles. The composition of the phospholipids of the *E*. *coli* extract has previously been investigated by NMR [25]. Here, an attempt was made to lower the particle size and dispersity (Đ) of the lipid solution prepared via the freeze–thaw treatment described in Section 3.3, with sonication and various sonication times, after which the lowered particle size and dispersity of the lipid vesicles were confirmed by DLS.

**Figure 6:**
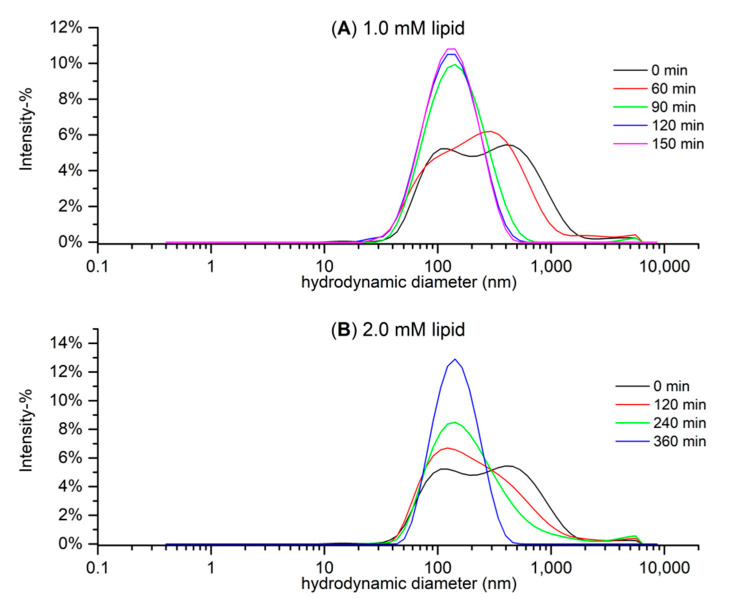
Hydrodynamic particle size distribution based on the intensity of (**A**) five 1.0 mM and (**B**) three 2.0 mM *E. coli* lipid extract solutions differing in the time they were sonicated after freeze–thaw treatment measured with dynamic light scattering (DLS). Note that the non-sonicated (0 min) sample of (**B**) was the 1.0 mM sample, as the non-sonicated version was not measured in 2.0 mM.

Preliminary tests were conducted with approximate lipid concentrations of 1 mM, and these DLS size distribution graphs are presented in Figure 6A. The lipid solutions became more monodisperse with longer sonication times, and the sample that was sonicated for 150 min had a main peak size (intensity-weighted hydrodynamic size) of 144.9 ± 3.9 nm and Đ of 0.206 ± 0.009 (mean ± SD). However, the concentration of the lipid solution had to be increased to 2 mM in order to receive sufficient heat in the ITC measurements. The higher lipid concentration seemed to slow down the lowering of the sample’s particle size and dispersity (Figure 6B) but after 360 min of sonication, the lipid sample had a main peak size of 155.8 ± 3.2 nm and Đ of 0.160 ± 0.014. In conclusion, we decided that the lipid samples that would be used in the ITC measurements would be subjected to the freeze–thaw treatment and 360 min of sonication.

**Figure 7:**
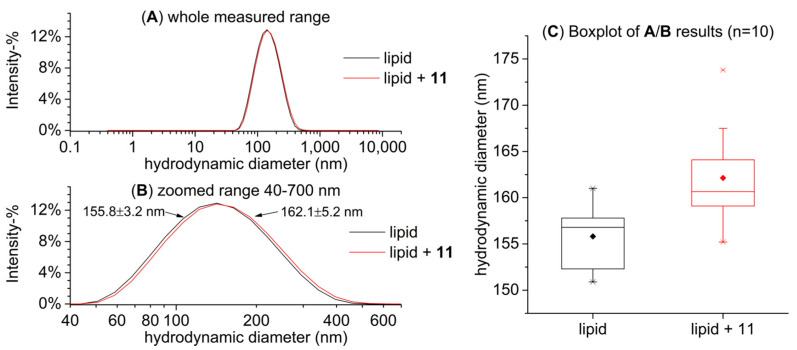
Hydrodynamic particle size distribution based on intensity of a 2 mM lipid solution sonicated for 360 min after freeze–thaw treatment alone and with the addition of pentagalloylglucose (**11**): (**A**) the whole measured range, (**B**) a zoomed range of 40–700 nm to highlight the difference between samples (mean ± SD, *n* = 10), and (**C**) boxplots of the main peak size for both samples (*n* = 10).

We also mimicked the conditions in the ITC sample cell during a typical measurement in order to see whether the HT–lipid interactions affected the size of the vesicles; i.e., we added HTs to a lipid solution that was measured in advance with DLS in order to see if this addition of HT had an effect on the particle size. The purpose was to verify that the addition of HT does not significantly perturb the structure of the formed vesicles, for example, by aggregation, and that the interactions we were measuring with ITC were in fact taking place with similar vesicles to those characterized with DLS. For this test, pentagalloylglucose (**11**) was utilized, which was added to the lipid solution in the same molar ratio that was present in the sample cell at the end of an ITC measurement after the full 20 injections. The results in Figure 7 demonstrate that the added pentagalloylglucose (**11**) did not cause clear detrimental changes, for example, vesicle breakdown, but in fact, the measured particle size was slightly larger (162.2 ± 5.2 nm) than that of the pure lipid sample (155.8 ± 3.2 nm). This small particle size increase makes sense when we consider that the added pentagalloylglucose (**11**) penetrates into the lipid bilayer increasing disorder in the otherwise ordered lipid bilayers and as a result possibly increasing the measured particle size [31]. Another potential cause for the increased particle size is that pentagalloylglucose molecules that are lodged partially in the bilayers can crosslink between multiple vesicles multidentately, which is a common phenomenon in tannin–protein interactions, and would thus increase the solution’s average particle size [33,34,35].

### 2.4. Stability Measurements of HTs in Buffer Solutions with UPLC-DAD-MS

**Figure 8:**
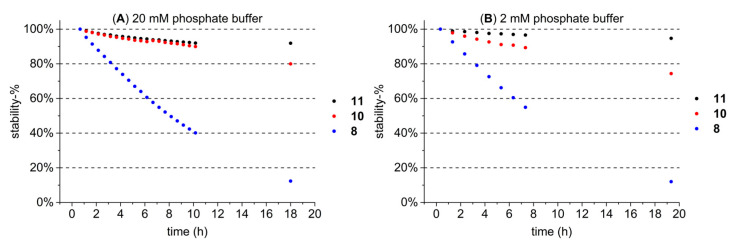
Stability of three HTs (pentagalloylglucose (**11**), tellimagrandin II (**10**), and vescalagin(**8**)) in 20 mM (**A**) and 2 mM (**B**) pH 7 sodiumphosphate buffer during approximately 20 h and presented as a stability-%, i.e., the percentage from the initial injection. The last data points were analyzed the next morning when the instrument was free, explaining the difference in the last data point’s time.

The stability of the studied HTs was monitored in the buffer solution (20 mM pH 7 sodiumphosphate buffer) that was used to prepare the lipid solution with three structurally different HTs (Figure 8A) and a diluted 2 mM version of the same buffer (Figure 8B). These three HTs were pentagalloylglucose (**11**), tellimagrandin II (**10**), and vescalagin (**8**); and they were selected to represent the HTs from the less stable acyclic ones (**8**) to more stable galloylglucoses (**11**) based on assumptions made on their stability in previous tests (data not shown) and literature [3,36]. The buffer was tested with a lower concentration to see if it would lower the rate at which the more unstable HTs degrade in the buffer, but the difference was not notable (Figure 8A,B). Stability measurements were done with a UPLC-DAD-MS instrument described in Section 3.6, and results were quantified from the UV trace at 280.0 ± 0.5 nm as peak areas.

Results showed that after approximately 10 h of incubation in the 20 mM buffer solution, the more stable HTs **11** and **10** still had 92% and 90% of their original concentrations remaining, respectively, whereas **8** had dissociated to only 40% of its original concentration. Based on these results, it was decided that each HT sample for each individual ITC measurement would be dissolved in the buffer solution right before the measurement to ensure minimal degradation of the HT. The total measurement time of one ITC measurement, including both the pre-equilibration and the actual measurement, was approximately 50 min, in which even the more unstable HTs did not degrade significantly; for example, after 60 min, 91% of **8** was still present (Figure 8A). It is also noteworthy that the HTs that dissociate easily, such as **8**, were also among the least active of all of the studied HTs; i.e., the effect of this small decrease in concentration on an already low detected lipid affinity was negligible.

## 3. Materials and Methods

### 3.1. Chemicals

Commercial *E. coli* total lipid extract was purchased from Avanti Polar Lipids (Alabaster, AL, USA) as a dry material, and it contained L-α-phosphatidylethanolamine (PE, 57.5 w-%), L-α-phosphatidylglycerol (PG, 15.1 w-%), and cardiolipin (CA, 9.8 w-%). The remaining 17.6 w-% consisted of an unidentified lipid according to the manufacturer. Sodium phosphate buffer reagents (NaH_2_PO_4_ and Na_2_HPO_4_) and LC-MS grade acetonitrile were purchased from Merck (KGaA, Darmstadt, Germany). LC-MS grade formic acid was purchased from VWR International (Fontenay-Sous-Bois, Paris, France). Type I ultrapure water was prepared with Merck Millipore Synergy UV system.

### 3.2. Isolation of Hydrolysable Tannins

The measured HTs were selected to represent the structural diversity of the compound group as extensively as possible, and emphasis was also placed on previous knowledge about the lipid interactions and the hydrophobicity of HTs [25,28]. In total, 24 HTs (Figure 1 and Figure 2) were used, and their purification from plants followed previously outlined methods [1,10,32,37,38,39,40]. Briefly, collected plant material was macerated in acetone and subsequently extracted 3–5 times with acetone/water (4:1, *v*/*v*). Extracts were then fractionated with Sephadex LH-20 gel material, followed by preparative and semipreparative HPLC fractionations. Purification stages and the final product purities were monitored with UPLC-DAD-ESI-MS, as discussed in Section 3.6 Purities of HTs, determined from the UV traces at 280.0 ± 0.5 nm by integrated peak areas, and original plant species, are presented in Supplementary Materials Table S1 with measured *m/z* values and errors for molecular ions. ^1^H-NMR spectra of the studied HTs were measured as discussed in Section 3.7 and are presented in Supplementary Materials Figures S6–S29.

### 3.3. Lipid Vesicle Preparation

The *E. coli* lipid vesicle solution was prepared via a similar freeze–thaw method as described in our previous work and adapted from Grélard et al. [25,41]. Briefly, a sufficient amount of the lipid extract for a 2 mM solution was dissolved in an Eppendorf with 20 mM pH 7 sodiumphosphate buffer. The solution was then frozen in liquid nitrogen, thawed in a 50 °C water bath, and then rigorously shaken in a vortex shaker. The process was repeated 4 times. The acquired lipid vesicle solution was then sonicated for approximately 6 h in 50 °C to modify the lipid vesicles to lower the dispersity and particle size.

### 3.4. ITC Measurements

The instrument utilized in all ITC measurements was a MicroCal iTC200 from Malvern Panalytical (Malvern, UK), and data handling was done with NanoAnalyze software version 3.12.0 from TA instruments (New Castle, DE, USA) and Origin 7 SR4 software version 7.0552 (B552) from OriginLab Corporation (Northampton, MA, USA) with an ITC extension. The sample and reference cell volumes were 200 µL, and the reference cell was filled with type I ultrapure H_2_O in all measurements. The stirring speed of the sample syringe during measurements was 750 rpm. The typical measurement was performed at 25 °C and consisted of an initial injection of 0.4 µL and 19 successive injections of 2.0 µL, with a 120 s equilibration period between injections. The initial injection was not included in the data analysis, in accordance with typical ITC procedures. Three types of control measurements were done to verify the actual amount of heat generated from the interactions between the titrated HT and the lipid vesicles. These control measurements were: (1) buffer titrated into lipid solution, (2) buffer titrated into buffer solution, and (3) HT titrated into buffer solution. Only the third one produced a substantial heat that was subtracted from each measurement and each tannin. Individual graphs of the observed heat rates of injections of the studied HTs plotted against the molar ratio of [HT]/[lipid] in the sample cell are presented in Figure S1. In addition, examples of the thermograms of the sample and control measurements are presented in Appendix A Figure A1, Figure A2 and Figure A3 and Supplementary Materials Figures S2–S5.

### 3.5. DLS Measurements

DLS measurements were performed with a Zetasizer Nano ZS instrument from Malvern Panalytical (Malvern, UK), and data were handled with Zetasizer software version 7.13. The instrument was operated at 633 nm, and back scattered light was recorded at an angle of 173°. The typical measurement consisted of 3 or 10 repetitions each with 13 size runs at 20 °C in a closed, disposable plastic cuvette. The lower number of repetitions was utilized while testing different lipid vesicle preparation methods and the higher number for determining the final lipid vesicles used in the ITC measurements.

### 3.6. UPLC-DAD-MS/MS Analyses

The stability of the studied HTs in different buffer solutions was tested and analyzed with an Acquity UPLC (Waters Corp., Milford, MA, USA) instrument consisting of a binary solvent manager, a sample manager, a column, and a diode array detector. The column utilized was an Aquity BEH phenyl column (2.1 × 100 mm, 1.7 µm; Waters Corp., Wexford, Ireland). The mobile phase consisted of acetonitrile (A) and 0.1% aqueous formic acid (B) with a constant flow rate of 0.5 mL/min with the following gradient: 0–0.5 min: 0.1% A; 0.5–5.0 min: 0.1–30% A (linear gradient); 5.0–6.0 min: 30–35% (linear gradient); 6.0–6.1 min: 35–90% A (linear gradient); 6.1–8.1 min: 90% A; 8.1–8.2 min: 90–0.1% A (linear gradient); and 8.2–9.5 min: 0.1% A. Column temperature was 40 °C. The UPLC was connected to a Xevo TQ triple-quadrupole mass spectrometer (Waters Corp., Milford, MA, USA) coupled to the UPLC via an ESI source. Injection volume was 5 µL and UV (λ = 190–500 nm) and MS data were recorded from injection until the end of the gradient. ESI source parameters were as follows: capillary voltage 1.8 kV, desolvation temperature 650 °C, source temperature 150 °C, and desolvation and cone gas (N_2_) flow rates 1000 and 100 L/h respectively. The mass spectrometer was operated in negative ionization and full scan spectra with an *m*/*z* range of 150–2000 and compound group-specific multiple-reaction monitoring methods were measured [42].

The stability of the HTs was determined from integrated UV peak areas at 280.0 ± 0.5 nm from injections done periodically either every 30 or 60 min, and the MS measurements were used to verify the identities of the compounds, and more importantly, to survey the degradation products formed during the buffer incubation.

Exact mass measurements for the purified HTs were done with an instrument with an identical UPLC and conditions as described above attached via a heated electrospray ionization (HESI) source to a quadrupole-Orbitrap mass spectrometer (QExactive^™^, Thermo Fisher Scientific GmbH, Bremen, Germany). The Orbitrap analyzer was operated in negative ionization with following source parameters: spray voltage, −3.0kV; sheath, auxiliary, and sheath gas (N_2_) flow rates, 60, 20, and 0, respectively; capillary temperature, +380 °C; in-source collision induced dissociation, 0 eV. Full scan spectra were recorded through the gradient with the analyzers mass range set to *m*/*z* 150–2250, automatic gain control to 3e6, and resolution to 70,000.

### 3.7. NMR Measurements

NMR measurements were performed with a Bruker AVANCE-III 600 MHz spectrometer equipped with a Prodigy TCI (inverted CryoProbe) cooled via liquid nitrogen or a Bruker AVANCE-III 500 MHz spectrometer equipped with a broad-band smart probe. For HT characterizations, typical ^1^H and ^13^C spectra were recorded, along with homo- and heteronuclear DQF-COSY, NOESY, TOCSY, HSQC, and HMBC 2D NMR experiments.

## 4. Conclusions

The utilization of ITC was an effective way to qualitatively study the interactions of biomimetic lipid vesicles and HTs and provided an excellent way to compare the strength of the interaction and the structural features of HTs to find the impactful functional groups. Even though ITC is generally considered to require large sample concentrations, we were able to make more definite structural comparisons than in the previous study, where we utilized HR-MAS NMR, in which weaker compounds produced even smaller signals than with ITC. Due to the smaller sample amounts and faster measurements, a larger set of HT structures could be compared. The HT–lipid interactions were exothermic, and some of them were in a similar range (up to −80–0 kJ/mol of injectant) to what has been previously reported for tannin–protein interactions. The following structural features of HTs were dominant for their lipid interactions. Free galloyl groups in the HTs increased their affinity for lipids, and HHDP groups were also observed to increase the interaction, but to a lesser extent than galloyl groups. Additionally, the flexibility and rotational freedom of the entire HT structure proved to be crucial. Additionally, the size of the HT molecule may play a role in the HT–lipid interactions if other structural features are excluded. The hydrophobicity of HTs does not alone determine its affinity for lipids. as moderately hydrophobic geraniin, chebulagic acid, and chebulinic acid showed weak affinities for lipids.

## Data Availability

The data presented in this study is available on request from the corresponding author.

## Supplementary Materials

The following supporting information can be downloaded at: https://www.mdpi.com/article/10.3390/molecules27103204/s1. Table S1: Molecular formula, UPLC retention times (min), calculated exact masses, mass error (ppm), and exact masses of the main ions, along with the purity of studied hydrolysable tannins. English and Latin names and plant parts used of the original plant material. Figure S1: Observed heat rates of injection of the studied HTs plotted against the molar ratio of HT/lipid in the sample cell. HTs presented in order of ascending molecular weight. Figure S2: Example thermograms of HT **1**–**7** sample (left) and control (right, HT titrated into buffer solution) measurements plotted against measurement time. Figure S3: Example thermograms of HT **8**–**14** sample (left) and control (right, HT titrated into buffer solution) measurements plotted against measurement time. Figure S4: Example thermograms of HT **15**–**21** sample (left) and control (right, HT titrated into buffer solution) measurements plotted against measurement time. Figure S5: Example thermograms of HT **22**–**24** sample (left) and control (right, HT titrated into buffer solution) measurements plotted against measurement time. Figures S6–S29: ^1^H-NMR spectra, peak coupling constants, chemical shifts, and multiplicities of HTs **1–24** measured in acetone-*d6* at 25 °C.
