# Supplementary material for: Interactions between Hydrolysable Tannins and Lipid Vesicles from Escherichia coli with Isothermal Titration Calorimetry
Source: Molecules. 2022 May 17;27(10):3204. doi: 10.3390/molecules27103204 (PMC9146631; doi:10.3390/molecules27103204)
Supplement: Supplementary file 1 [file molecules-27-03204-s001.zip › molecules-1684082-supplementary.pdf]

Supplementary Materials

Interactions Between Hydrolysable Tannins and Lipid Vesicles from *Escherichia coli* with Isothermal Titration Calorimetry

Valtteri Virtanen [1]*, Rebecca J. Green [2] and Maarit Karonen[1]

[1] Natural Chemistry Research Group, Department of Chemistry, University of Turku, Turku FI-20014, Finland; vtjvir@utu.fi (V.V); maarit.karonen@utu.fi (M.K.)

[2] School of Chemistry, Food and Pharmacy, University of Reading, Whiteknights, P.O. Box 224, Reading RG6 6AP, United Kingdom; rebecca.green@reading.ac.uk

* Correspondence: vtjvir@utu.fi; Tel.: Tel.: +358-29-450-3205 (V.V.)

Table of Contents

Table S1. Molecular formula, UPLC retention times (min), calculated exact masses, mass error (ppm) and exact masses of the main ions and the purity of studied hydrolysable tannins. English and Latin names and plant part used of the original plant material. (3)

Figure S1. Observed heat rates of injection of the studied HTs as a function of the molar ratio ([HT]/[lipid]) in the sample cell. (4)

Figure S2. Example thermograms of HTs 1–7 sample (left) and control (right, HT titrated into buffer solution) measurements plotted against measurement time with the y-axis normalized according to the maximum of each set (sample/control). (5)

Figure S3. Example thermograms of HTs 8–14 sample (left) and control (right, HT titrated into buffer solution) measurements plotted against measurement time with the y-axis normalized according to the maximum of each set (sample/control). (6)

Figure S4. Example thermograms of HTs 15–21 sample (left) and control (right, HT titrated into buffer solution) measurements plotted against measurement time with the y-axis normalized according to the maximum of each set (sample/control). (7)

Figure S5. Example thermograms of HTs 22–24 sample (left) and control (right, HT titrated into buffer solution) measurements plotted against measurement time with the y-axis normalized according to the maximum of each set (sample/control). (8)

Figure S6. ^{1}H-NMR spectrum and peak assignations of corilagin (1). (9)

Figure S7. ^{1}H-NMR spectrum and peak assignations of isostrictinin (2). (10)

Figure S8. ^{1}H-NMR spectrum and peak assignations of strictinin (3). (11)

Figure S9. ^{1}H NMR spectrum and peak assignations of 1,2,6-tri-O-galloyl-β-D-glucose (4). (12)

Figure S10. ^{1}H NMR spectrum and peak assignations of pedunculagin (5). (13)

Figure S11. ^{1}H NMR spectrum and peak assignations of tellimagrandin I (6). (14)

Figure S12. ^{1}H-NMR spectrum and peak assignations of 1,2,3,6/1,2,4,6-tetra-*O*-galloyl-β-*D*-glucose (7). (15)

Figure S13. ^{1}H-NMR spectrum and peak assignations of vescalagin (8). (16)

Figure S14. ^{1}H-NMR spectrum and peak assignations of casuarictin (9). (17)

Figure S15. ^{1}H-NMR spectrum and peak assignations of tellimagrandin II (10). (18)

Figure S16. ^{1}H-NMR spectrum and peak assignations of 1,2,3,4,6-penta-*O*-galloyl-β-*D*-glucose (11). (19)

Figure S17. ^{1}H-NMR spectrum and peak assignations of geraniin (12). (20)

Figure S18. ^{1}H-NMR spectrum and peak assignations of chebulagic acid (13). (21)

Figure S19. ^{1}H-NMR spectrum and peak assignations of chebulinic acid (14). (22)

Figure S20. ^{1}H-NMR spectrum and peak assignations of punicalagin (15). (23)

Figure S21. ^{1}H-NMR spectrum and peak assignations of oenothein B (16). (24)

Figure S22. ^{1}H-NMR spectrum and peak assignations of rugosin E (17). (25)

Figure S23. ^{1}H-NMR spectrum and peak assignations of agimoniin (18). (26)

Figure S24. ^{1}H-NMR spectrum and peak assignations of sanguiin H-6 (19). (27)
Figure S25. ^{1}H-NMR spectrum and peak assignations of gemin A (20). (28)
Figure S26. ^{1}H-NMR spectrum and peak assignations of rugosin D (21). (29)
Figure S27. ^{1}H-NMR spectrum and peak assignations of oenothein A (22). (30)
Figure S28. ^{1}H-NMR spectrum and peak assignations of lambertianin C (23). (31)
Figure S29. ^{1}H-NMR spectrum and peak assignations of rugosin G (24). (32)

Table S**1**. Molecular formula, UPLC retention times (min), calculated exact masses, mass error (ppm) and exact masses of the main ions and the purity of studied hydrolysable tannins. English and Latin names and plant part used of the original plant material.

#	Hydrolysable tannin	Molecular formula	Retention time (min)[*]	$M_{calculated}$	Error (ppm)[**]	Observed *m/z*		Purity-%	Plant origin		
						$[M-H]^-$	$[M-2H]^{2-}$		English name	Latin name	Plant part
1	corilagin	$C_{27}H_{22}O_{18}$	3.27	634.08062	-0.927	633.07275	-	98 %	Black myrabolan	*Terminalia chebula*	leaf
2	isostrictinin	$C_{27}H_{22}O_{18}$	2.55	634.08062	-0.943	633.07274	-	93 %	Sea buckthorn	*Hippophae rhamnoides*	leaf
3	strictinin	$C_{27}H_{22}O_{18}$	3.11	634.08062	-0.832	633.07281	-	99 %	Sea buckthorn	*Hippophae rhamnoides*	leaf
4	1,2,6-tri-*O*-galloyl-β-D-glucose	$C_{27}H_{24}O_{18}$	3.39	636.09627	-0.956	635.08838	-	93 %	Sea buckthorn	*Hippophae rhamnoides*	leaf
5	pedunculagin	$C_{34}H_{24}O_{22}$	2.53/2.85	784.07593	-0.722	783.06808	-	99 %	Silverweed	*Argentina anserina*	leaf
6	tellimagrandin I	$C_{34}H_{26}O_{22}$	3.10/3.33	786.09158	-0.771	785.08369	-	95 %	Meadowsweet	*Filipendula ulmaria*	flower
7	1,2,3,6/1,2,4,6-tetra-*O*-galloyl-β-D-glucose[b]	$C_{34}H_{28}O_{22}$	4.02/4.08	788.10723	-0.845	787.09928	-	24 % / 71 %	Norway maple	*Acer platanoides*	leaf
8	vescalagin	$C_{41}H_{26}O_{26}$	2.23	934.07124	-0.744	933.06326	-	97 %	English oak	*Quercus robur*	acorn
9	casuarictin	$C_{41}H_{28}O_{26}$	3.82	936.08689	-0.421	935.07921	-	98 %	Meadowsweet	*Filipendula ulmaria*	flower
10	tellimagrandin II	$C_{41}H_{30}O_{26}$	4.00	938.10254	-0.218	937.09505	-	90 %	Meadowsweet	*Filipendula ulmaria*	flower
11	1,2,3,4,6-penta-*O*-galloyl-β-D-glucose[***]	$C_{41}H_{32}O_{26}$	4.38	940.11819	-0.526	939.11041	-	99 %	-	-	-
12	geraniin	$C_{41}H_{28}O_{27}$	3.41	952.08181	-0.976	951.07359	-	85 %	Wood cranesbill	*Geranium sylvaticum*	leaf
13	chebulagic acid	$C_{41}H_{30}O_{27}$	3.87	954.09746	-0.796	953.08941	-	96 %	Black myrabolan	*Terminalia chebula*	leaf
14	chebulinic acid	$C_{41}H_{32}O_{27}$	4.28	956.11311	-0.909	955.10495	-	88 %	Black myrabolan	*Terminalia chebula*	leaf
15	punicalagin	$C_{48}H_{28}O_{30}$	2.72/3.05	1084.06655	-0.153	1083.05848	541.02591	98 %	Black myrabolan	*Terminalia chebula*	leaf
16	oenothein B	$C_{68}H_{48}O_{44}$	2.95/3.24	1568.15186	-0.913	1567.14170	783.06793	93 %	Willowherb	*Chamaenerion angustifolium*	leaf
17	rugosin E	$C_{75}H_{54}O_{48}$	3.86/3.90	1722.17847	0.094	1721.17012	860.08203	91 %	Meadowsweet	*Filipendula ulmaria*	flower
18	agrimoniin	$C_{82}H_{54}O_{52}$	4.16	1870.15813	-0.341	1869.14675	934.07146	98 %	Silverweed	*Argentina anserina*	leaf
19	sanguiin H-6	$C_{82}H_{54}O_{52}$	3.86	1870.15813	-0.363	1869.14661	934.07144	97 %	Raspberry	*Rubus idaeus*	leaf
20	gemin A	$C_{82}H_{56}O_{52}$	4.07	1872.17378	-0.389	1871.16295	935.07924	97 %	herb Bennet	*Geum urbanum*	leaf
21	rugosin D	$C_{82}H_{58}O_{52}$	4.22	1874.18943	-0.309	-	936.08714	91 %	Meadowsweet	*Filipendula ulmaria*	flower
22	oenothein A	$C_{102}H_{72}O_{66}$	3.28	2352.22779	-0.158	-	1175.10642	85 %	Willowherb	*Chamaenerion angustifolium*	leaf
23	lambertianin C	$C_{123}H_{78}O_{78}$	3.81	2802.21372	-1.459	-	1401.10535	98 %	Raspberry	*Rubus idaeus*	leaf
24	rugosin G	$C_{123}H_{86}O_{78}$	4.18	2810.27632	-2.264	-	1404.12769	90 %	Meadowsweet	*Filipendula ulmaria*	flower

[*]UPLC retention time from an analysis performed with a gradient described in the article section 3.5. [**] Mass error presented for the underlined ion. [***]Pentagalloylglucose prepared from tannic acid via methanolysis.

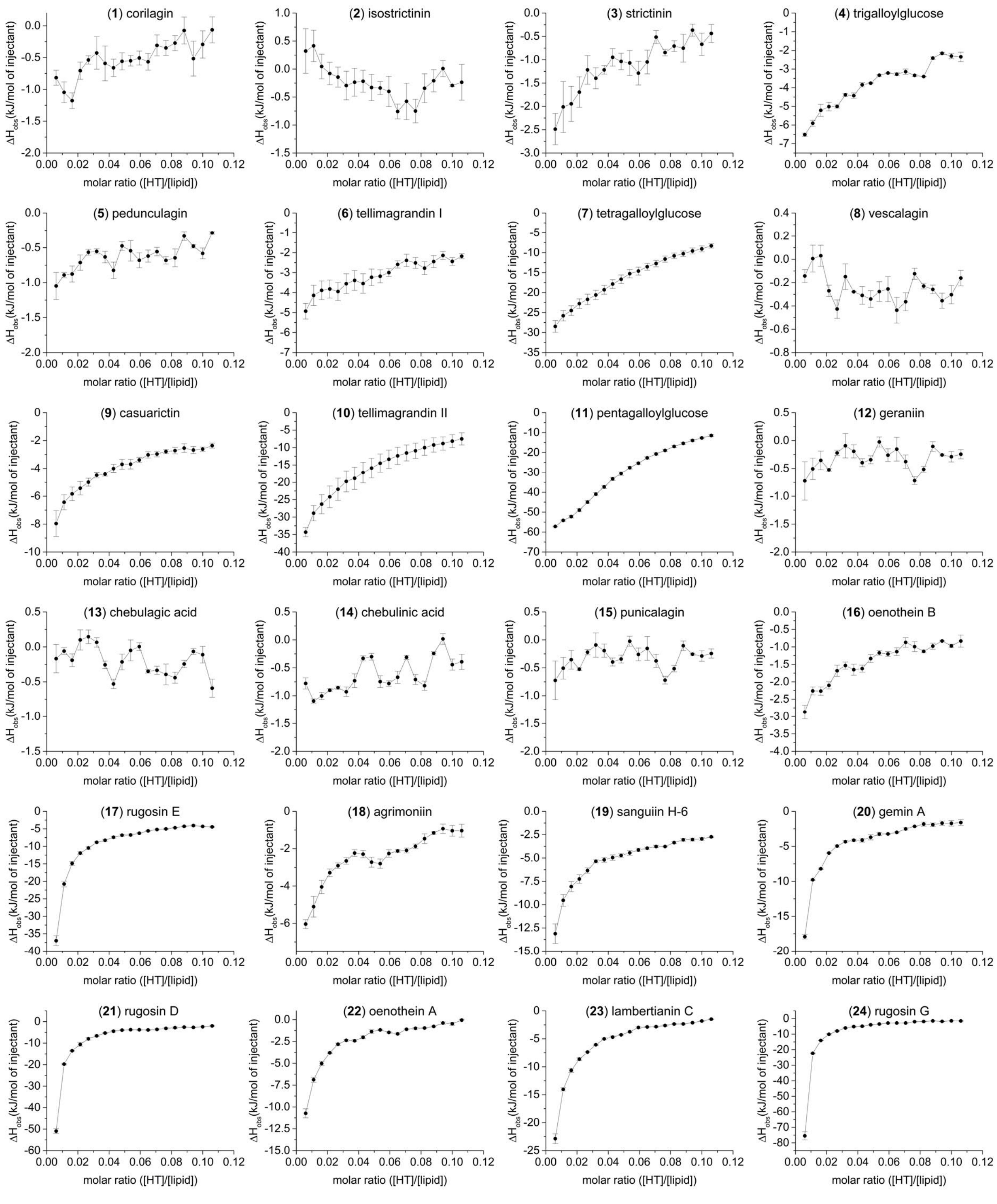

Figure S1. Observed heat rates of injection of the studied HTs as a function of the molar ratio ([HT]/[lipid]) in the sample cell. Heat rates presented as kJ/mol of injectant with average values and standard error, n=3. HTs are presented in the order of ascending molecular weight. For the structures of HTs refer to Figures 1 and 2 in the article.

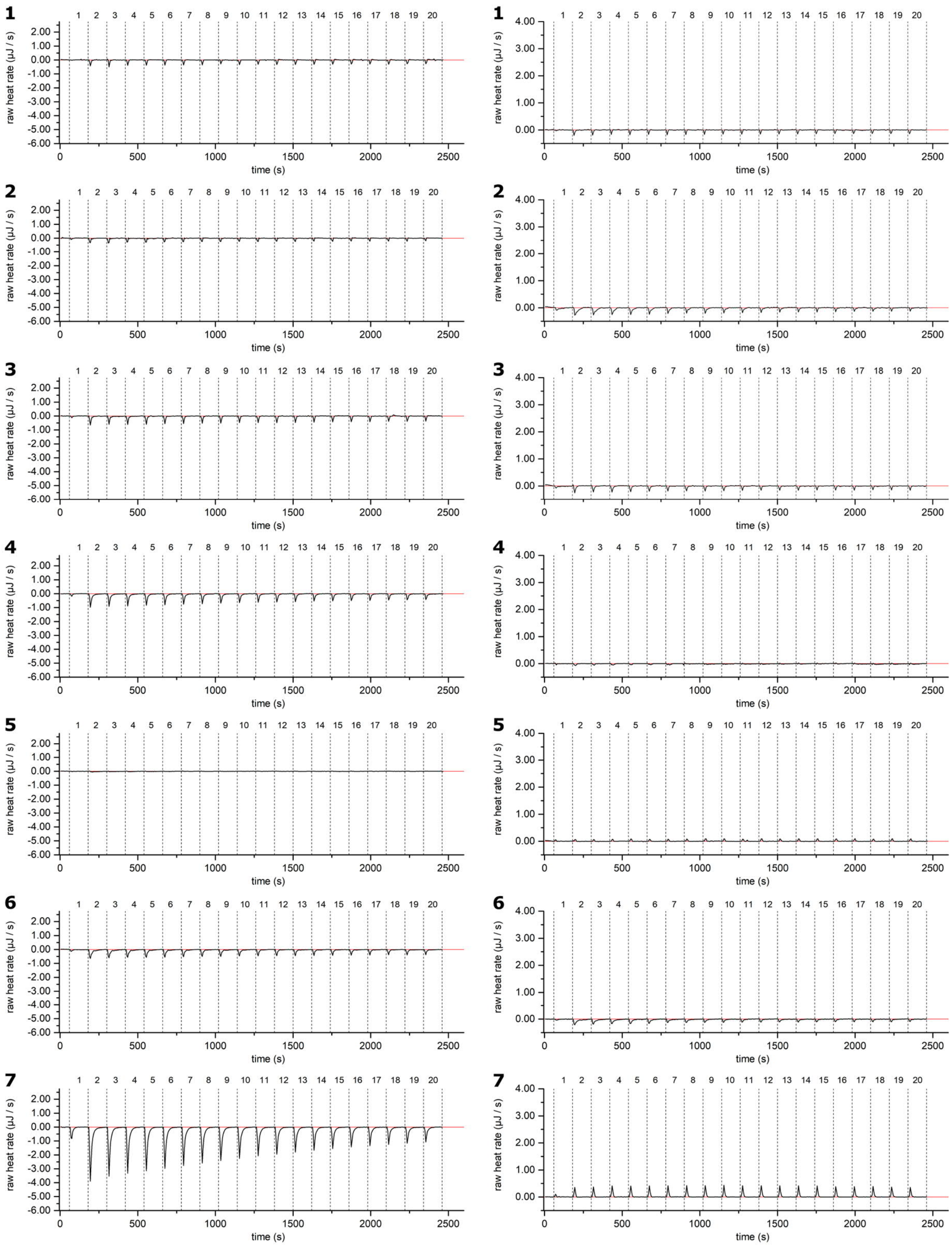

Figure S2. Example thermograms of HTs 1–7 sample (left) and control (right, HT titrated into buffer solution) measurements plotted against measurement time with the y-axis normalized according to the maximum of each set (sample/control). Corrected baseline presented as red line and injections separated with dashed lines. HTs presented in order of ascending molecular weight. For the structures and numbering of HTs refer to Figures 1 and 2 in the article.

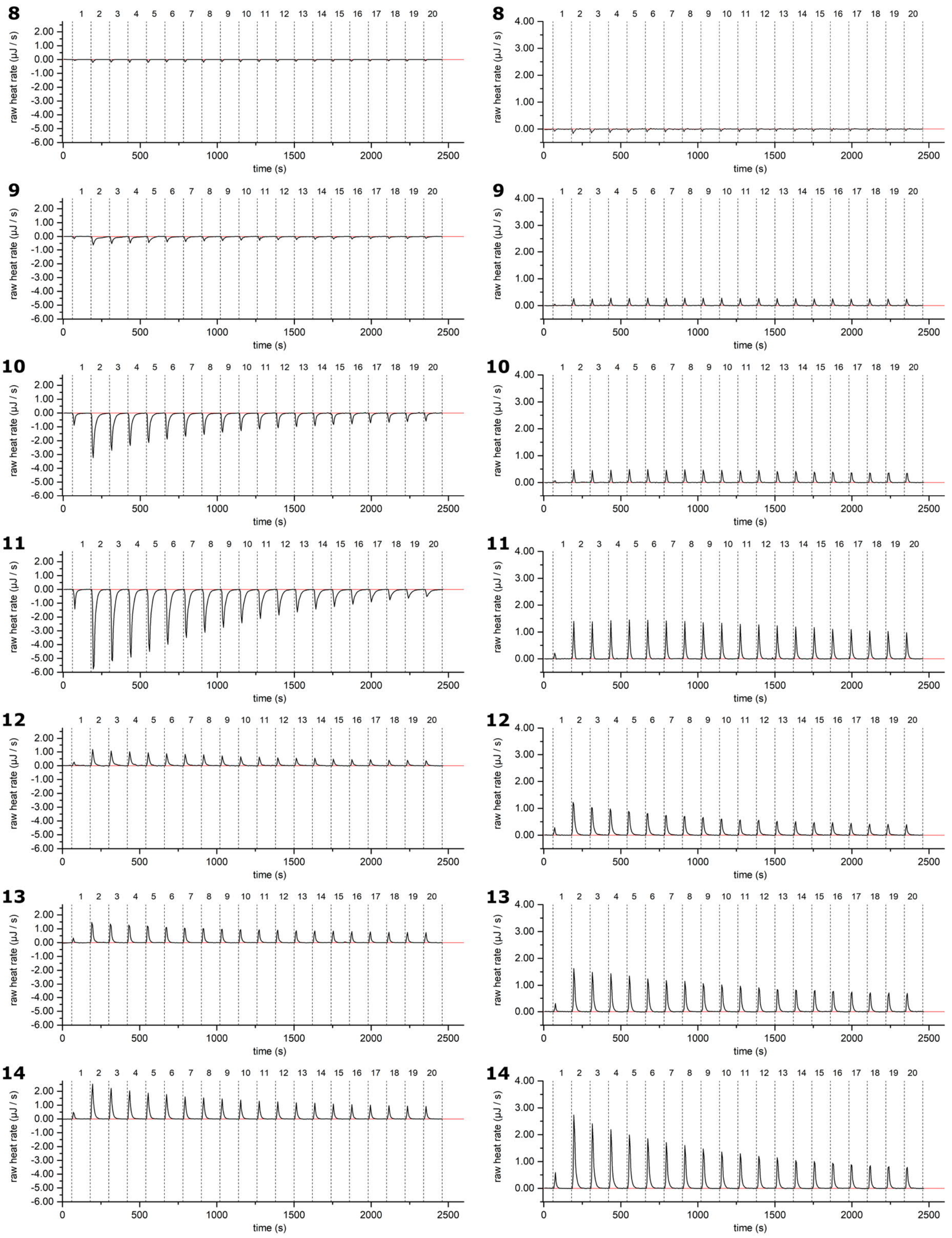

Figure S3. Example thermograms of HTs 8–14 sample (left) and control (right, HT titrated into buffer solution) measurements plotted against measurement time with the y-axis normalized according to the maximum of each set (sample/control). Corrected baseline presented as red line and injections separated with dashed lines. HTs presented in order of ascending molecular weight. For the structures and numbering of HTs refer to Figures 1 and 2 in the article.

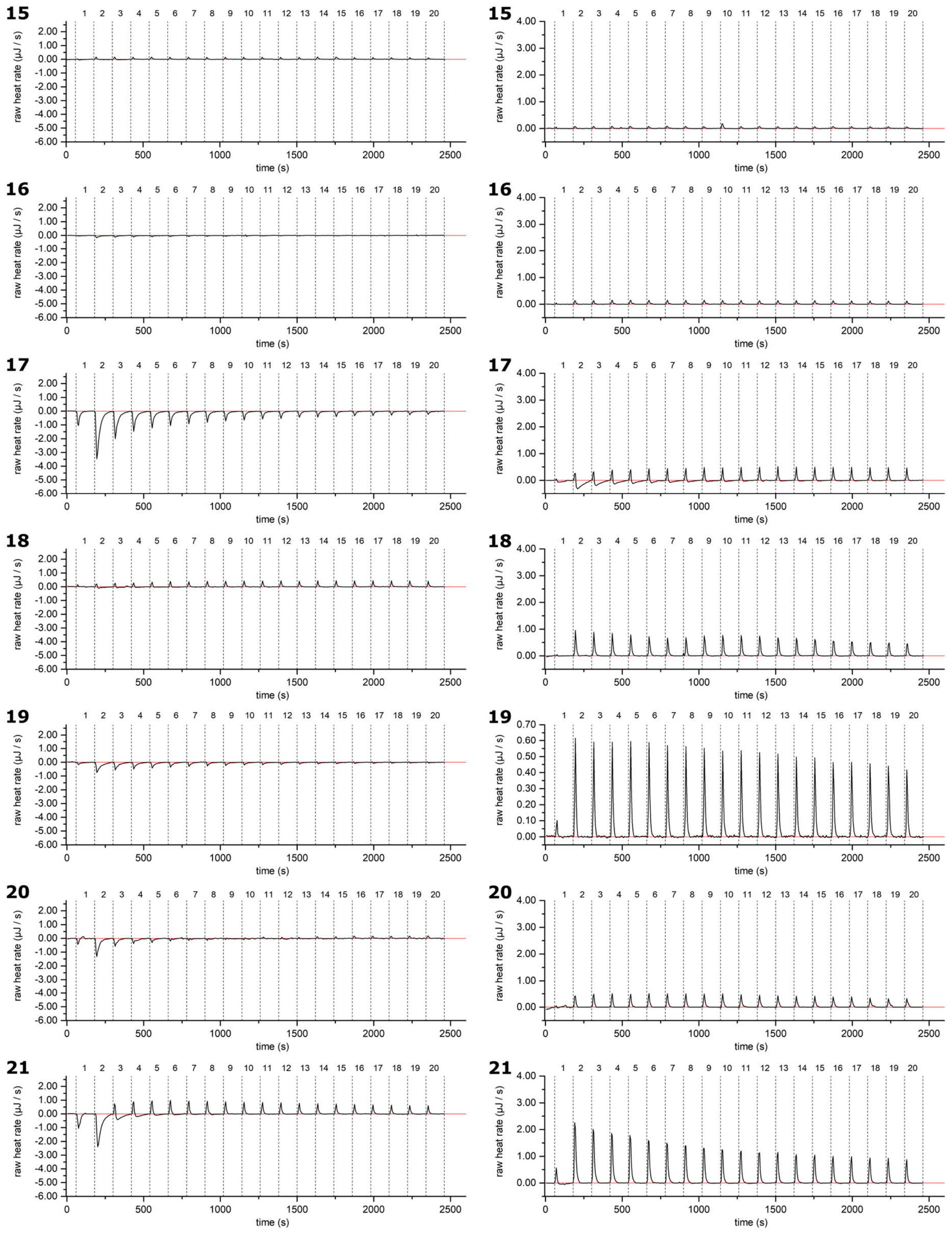

Figure S4. Example thermograms of HTs 15–21 sample (left) and control (right, HT titrated into buffer solution) measurements plotted against measurement time with the y-axis normalized according to the maximum of each set (sample/control). Corrected baseline presented as red line and injections separated with dashed lines. HTs presented in order of ascending molecular weight. For the structures and numbering of HTs refer to Figures 1 and 2 in the article.

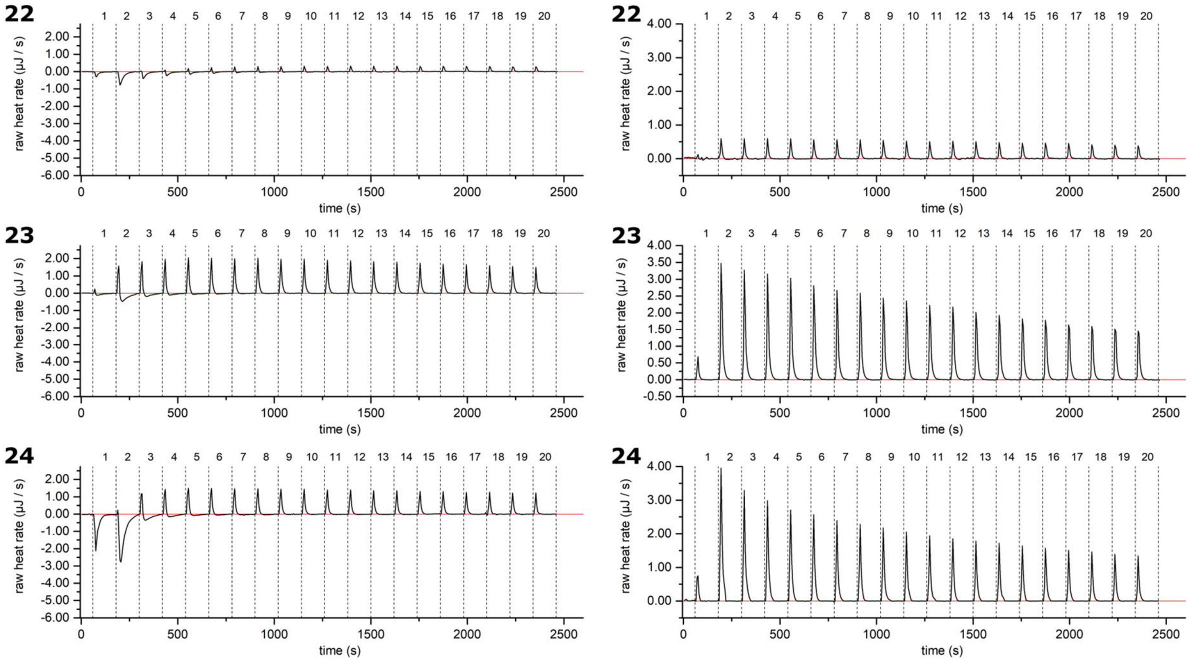

Figure S5. Example thermograms of HTs 22–24 sample (left) and control (right, HT titrated into buffer solution) measurements plotted against measurement time with the y-axis normalized according to the maximum of each set (sample/control). Corrected baseline presented as red line and injections separated with dashed lines. HTs presented in order of ascending molecular weight. For the structures and numbering of HTs refer to Figures 1 and 2 in the article.

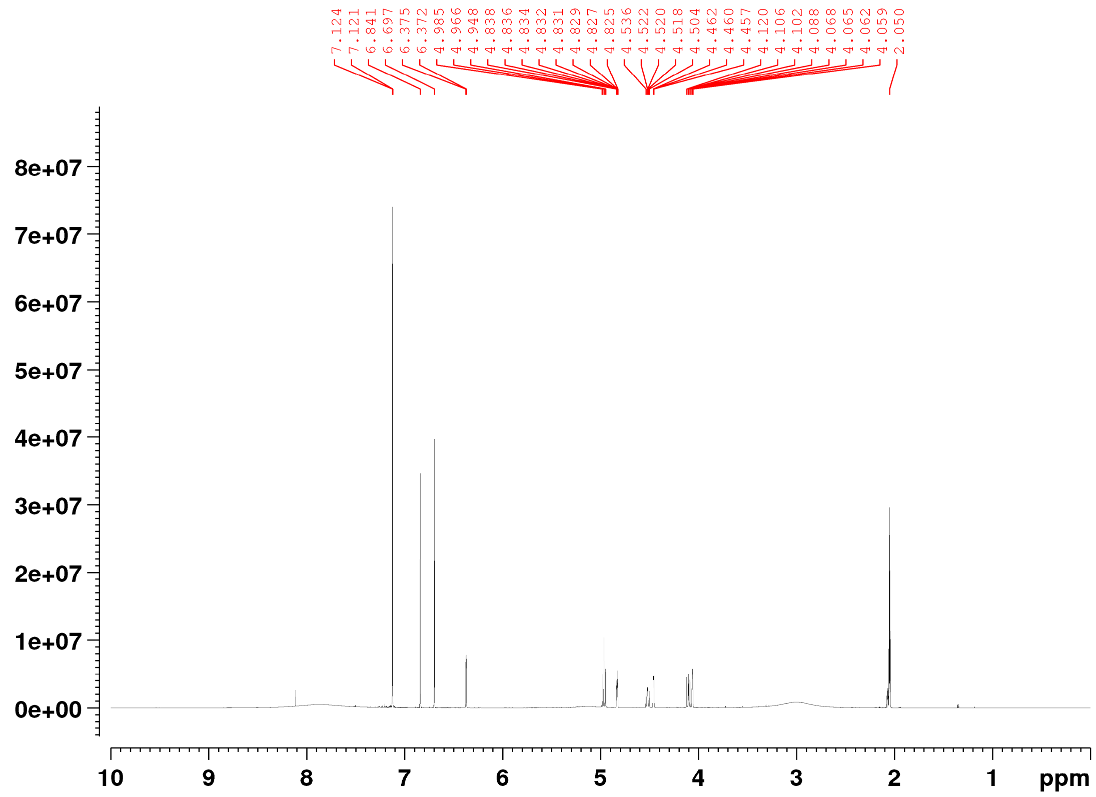

Figure S6. ^{1}H-NMR spectrum and peak assignations of corilagin (1). ^{1}H NMR (600 MHz, acetone-*d6*) δ 4.06 (br s, 1H), δ 4.10 (dd, 1H, J=8.1, 11.0 Hz), δ 4.46 (br s, 1H), δ 4.52 (m, 1H), δ 4.83 (m, 1H), δ 4.97 (t, 1H, J=11.0 Hz), δ 6.37 (d, 1H, J=2.0 Hz), δ 6.70 (s, 1H), δ 6.84 (s, 1H), δ 7.12 (s, 2H).

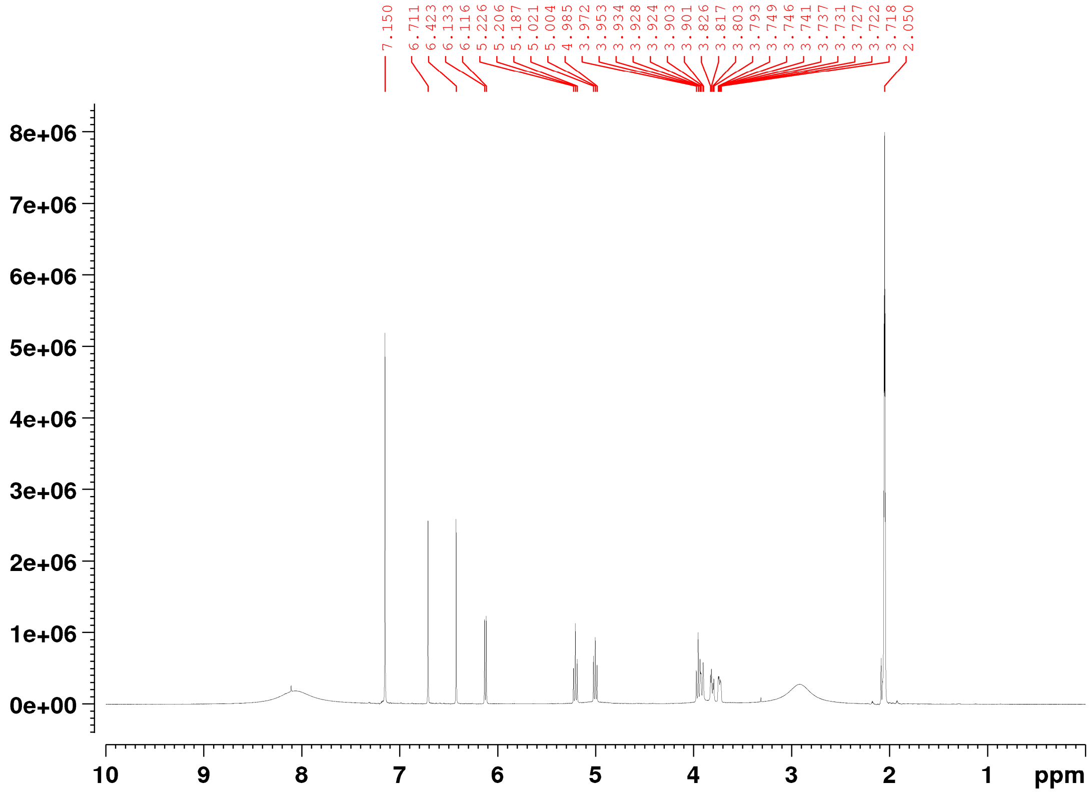

Figure S7. ^{1}H-NMR spectrum and peak assignations of isostrictinin (2). ^{1}H NMR (500 MHz, acetone-*d6*) δ 3.74 (ddd, 1H, J=1.5, 4.0, 9.4 Hz), δ 3.81 (dd, 1H, J=4.6, 12.0 Hz), δ 3.91 (dd, 1H, J=1.8, 12.2 Hz), δ 3.95 (t, 1H, J=9.6 Hz), δ 5.00 (t, 1H, J=9.1 hz), δ 5.21 (t, 1H, J=9.5 Hz), δ 6.12 (d, 1H, J=8.5 Hz), δ 6.42 (s, 1H), δ 6.71 (s, 1H), δ 7.15 (s, 2H).

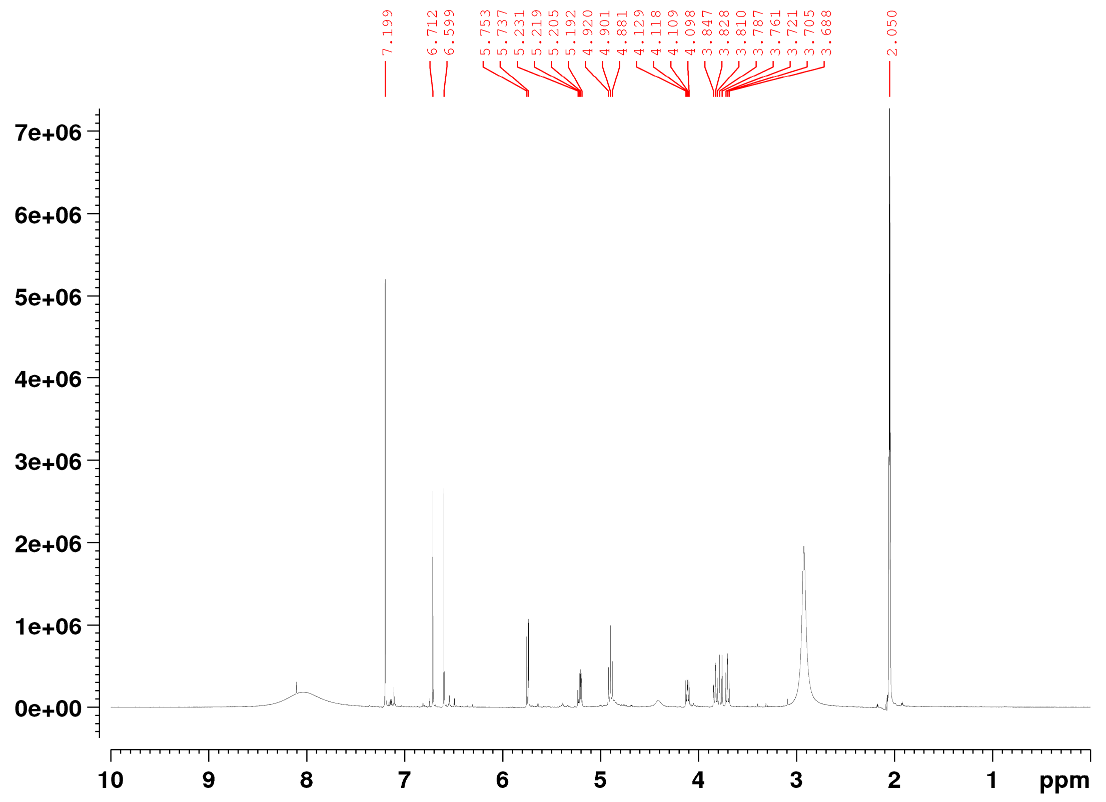

Figure S8. ^{1}H-NMR spectrum and peak assignations of strictinin (3). ^{1}H NMR (500 MHz, acetone-*d6*) δ 3.70 (t, 1H, J=8.5 Hz), δ 3.78 (d, 1H, J=13.0 Hz), δ 3.83 (d, 1H, J=9.3 Hz), δ 4.11 (dd, 1H, J=5.7, 9.9 Hz), δ 4.90 (t, 1H, J=9.8 Hz), δ 5.21 (dd, 1H, J=6.4, 13.3 Hz), δ 5.74 (d, 1H, J=8.1 Hz), δ 6.60 (s, 1H), δ 6.71 (s, 1H), δ 7.20 (s, 1H).

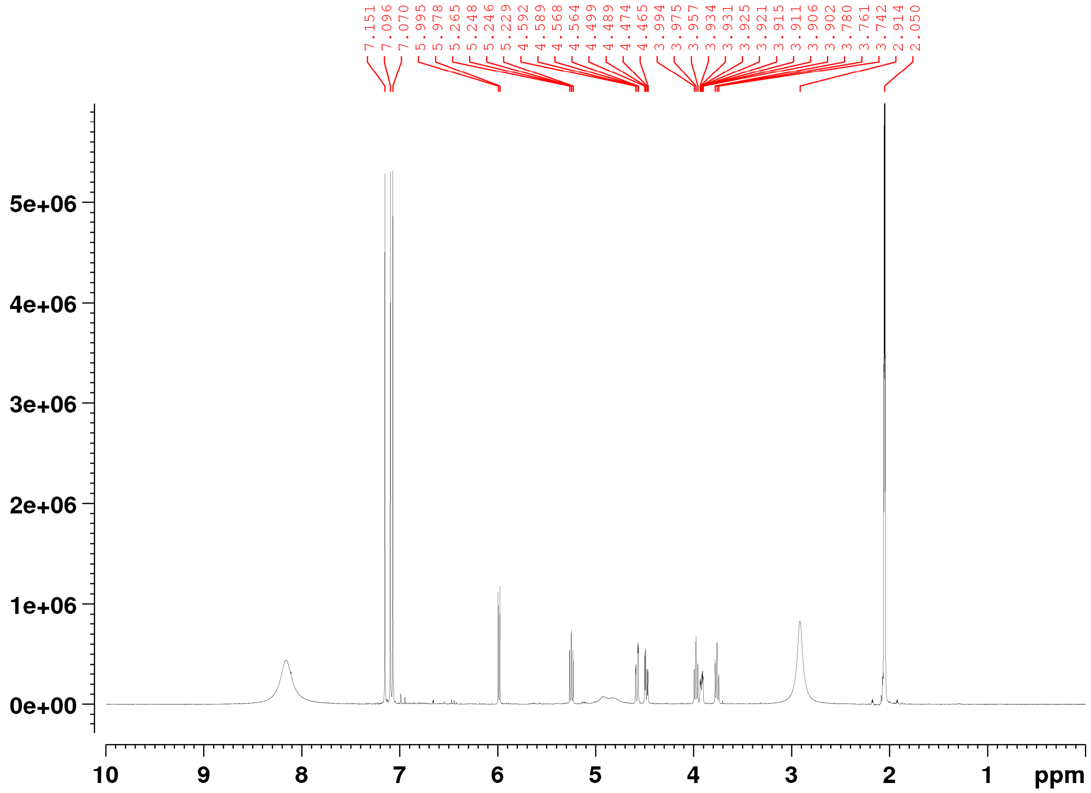

Figure S9. ^{1}H NMR spectrum and peak assignations of 1,2,6-tri-O-galloyl-β-D-glucose (4). ^{1}H NMR (500 MHz, acetone-*d6*) δ 3.76 (t, 1H, J=9.3 Hz), δ 3.92 (ddd, 1H, J=1.9, 4.6, 9.8 Hz), δ 3.98 (t, 1H, J=9.2 Hz), δ 4.48 (dd, 1H, J=4.6, 12.1 Hz), δ 4.58 (dd, 1H, J=1.8, 12.1 Hz), δ 5.25 (dd, 1H, J=8.6, 9.4 Hz), δ 5.99 (d, 1H, J=8.4 Hz), δ 7.07 (s, 1H), δ 7.10 (s, 1H), δ 7.15 (s, 1H).

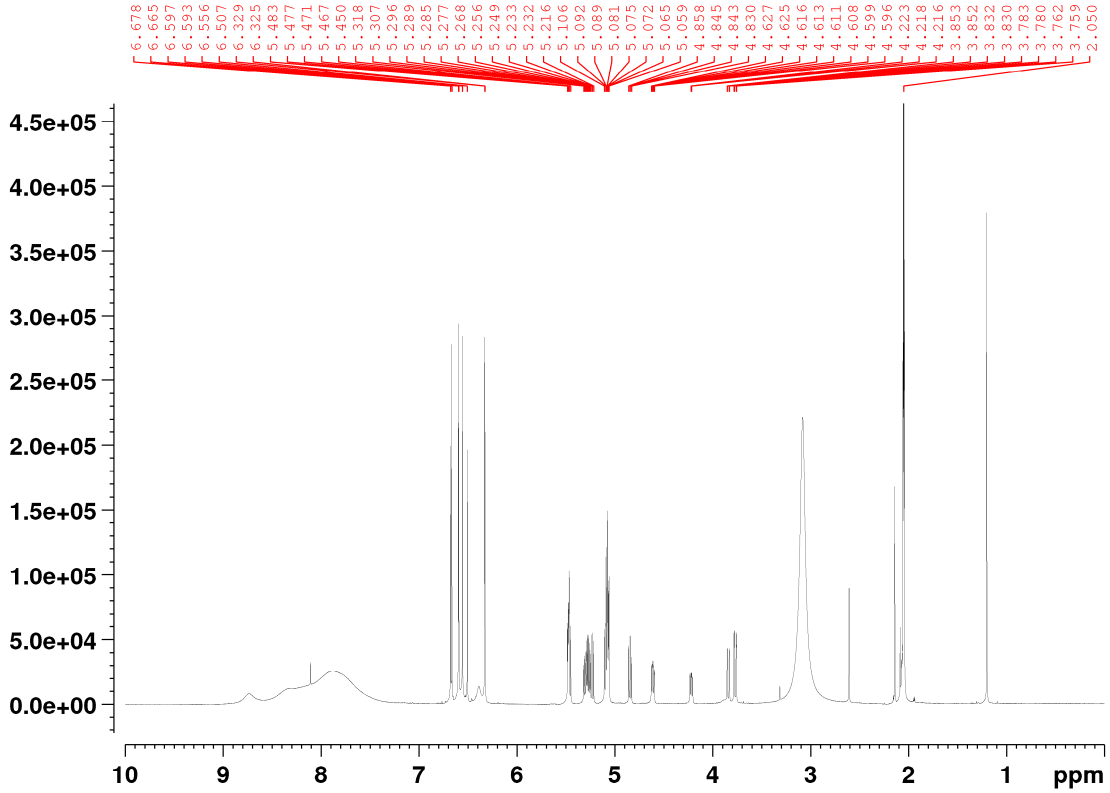

Figure S10. ^{1}H NMR spectrum and peak assignations of pedunculagin (5). Two epimers due to anomeric glucose C1 (α:β, 1.0:0.7). ^{1}H NMR (600 MHz, acetone-*d6*) δ 3.77 (dd, 1H, J=1.7, 12.8 Hz, α), δ 3.84 (dd, 1H, J=1.0, 12.9 Hz, β), δ 4.22 (ddd, 1H, J=1.2, 6.8, 9.8 Hz, β), δ 4.61 (ddd, 1H, J=1.7, 7.1, 10.0 Hz, α), δ 4.84 (dd, 1H, J=8.2, 9.0 Hz, β), δ 5.08 (m, 4H, both), δ 5.27 (m, 3H, both), δ 5.47 (m, 2H, both), δ 6.325 (s, 1H, β), δ 6.329 (s, 1H, α), δ 6.51 (s, 1H, β), δ 6.56 (s, 1H, α), δ 6.593 (s, 1H, β), δ 6.597 (s, 1H, α), δ 6.66 (s, 1H, α), δ 6.68 (s, 1H, β).

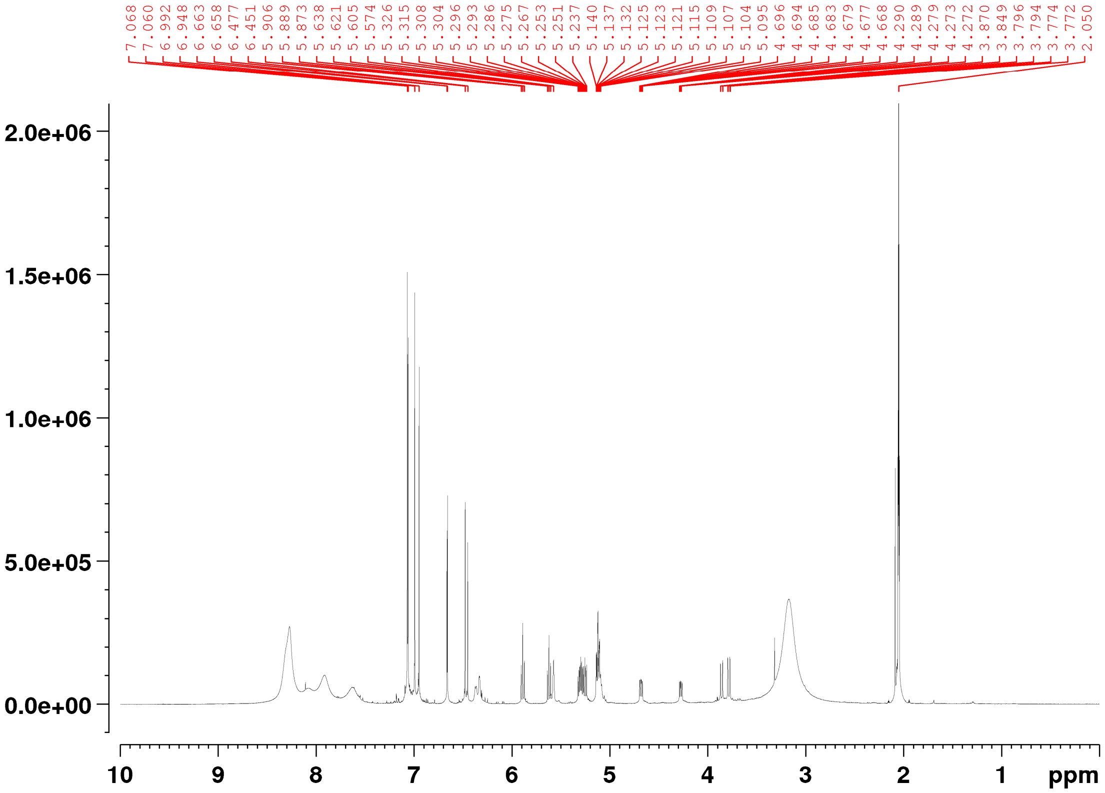

Figure S11. ^{1}H NMR spectrum and peak assignations of tellimagrandin I (6). Two epimers due to anomeric glucose C1 (α: β, 1.0:0.8). ^{1}H NMR (600 MHz, acetone-*d6*) δ 3.78 (dd, 1H, J=1.4, 13.1 Hz, α), δ 3.86 (br d, 1H, J=13.1 Hz, β), δ 4.28 (ddd, 1H, J=0.8, 6.6, 10.0 Hz, β), δ 4.68 (ddd, 1H, J=1.0, 6.7, 10.2 Hz, α), δ 5.11 (m, 4H, both), δ 5.28 (m, 3H, both), δ 5.57 (br s, 1H, α), δ 5.62 (t, 1H, J=9.8 Hz β), δ 5.89 (m, 1H, J= 10.0 α), δ 6.45 (s, 1H, β), δ 6.48 (s, 1H, α), δ 6.658/6.663 (br s/br s, 2H, both), δ 6.95 (s, 2H, β), δ 7.00 (s, 2H, α), δ 7.06 (s, 2H, β), δ 7.07 (s, 2H, α).

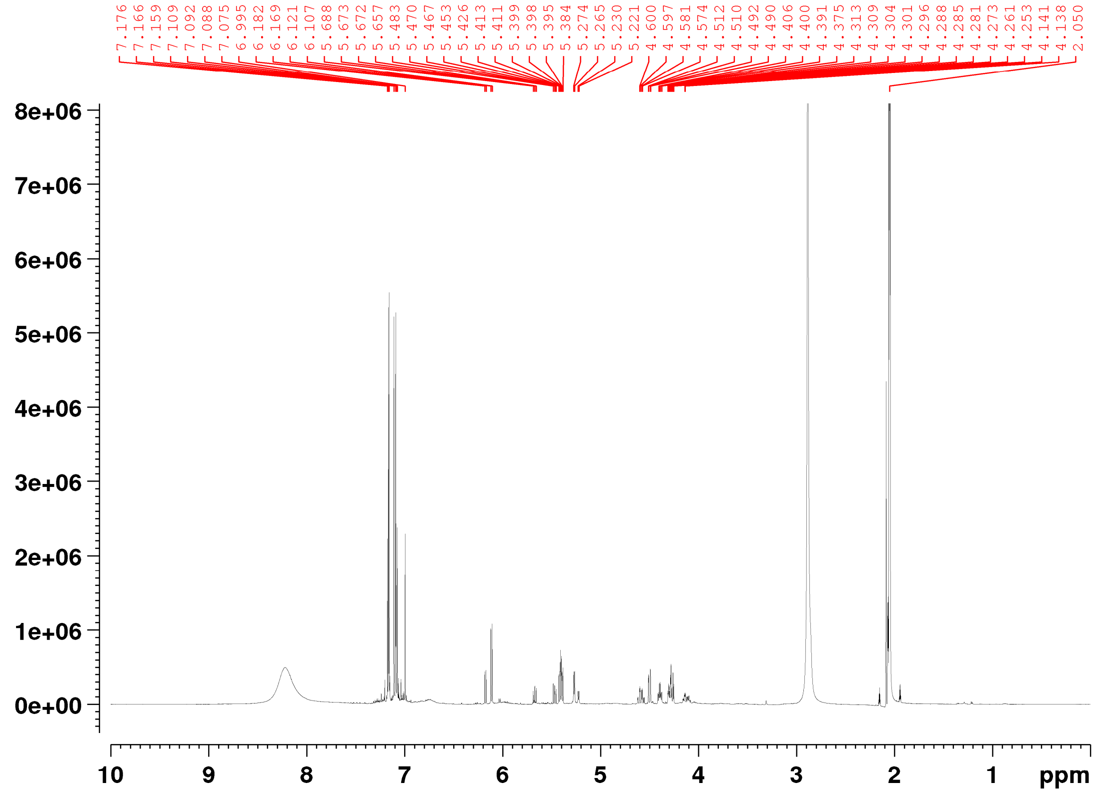

Figure S12. ^{1}H-NMR spectrum and peak assignations of 1,2,3,6/1,2,4,6-tetra-*O*-galloyl-β-D-glucose (7). 1,2,3,6-tetra-*O*-galloyl-β-D-glucose: ^{1}H NMR (600 MHz, acetone-*d6*) δ 4.11 (dd, 1H, J=5.6, 9.0 Hz), δ 4.13 (ddd, 1H, J=2.1, 4.1, 9.8 Hz), δ 4.57 (dd, 1H, J=4.3, 12.2 Hz), δ 4.61 (dd, 1H, J=2.0, 12.2 Hz), δ 5.47 (dd, 1H, J=8.4, 9.9), δ 5.67 (dd, 1H, J=8.9, 9.8), δ 6.18 (d, 1H, J=8.3), δ 7.00 (s, 2H), δ 7.08 (s, 2H), δ 7.09 (s, 2H), δ 7.18 (s, 2H). 1,2,4,6-tetra-O-galloyl-β-D-glucose: ^{1}H NMR (600 MHz, acetone-*d6*) δ 4.27 (dd, 1H, J=4.8, 12.0 Hz), δ 4.31 (m, 1H), δ 4.39 (td, 1H, J=5.5, 9.4 Hz), δ 4.50 (dd, 1H, J=1.4, 11.9 Hz), δ 5.40 (dd, 1H, J=8.5, 9.5 Hz), δ 5.41 (t, 1H, J=9.5 Hz), δ 6.11 (d, 1H, J=8.4 Hz), δ 7.09 (s, 2H), δ 7.11 (s, 2H), δ 7.16 (s, 2H), δ 7.17 (s, 2H).

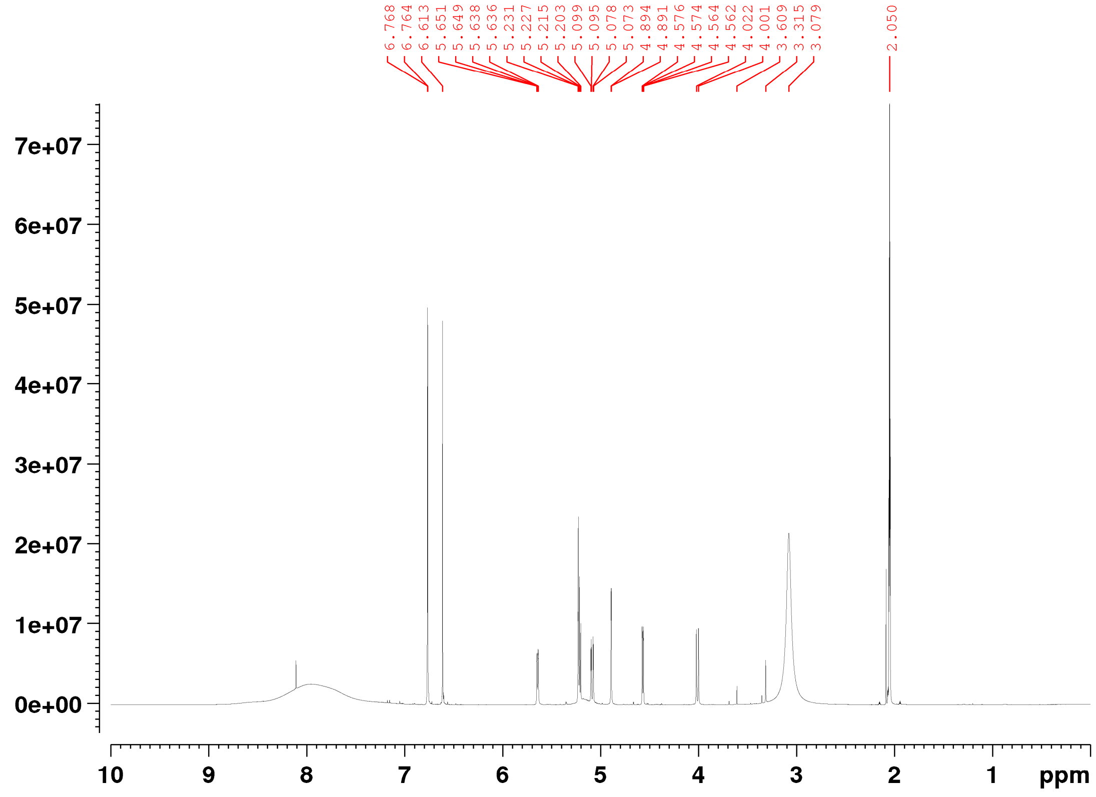

Figure S13. ^{1}H-NMR spectrum and peak assignations of vescalagin (8). ^{1}H NMR (600 MHz, acetone-*d6*) δ 4.02 (d, 1H, J=12.4 Hz), δ 4.57 (dd, 1H, J=1.4, 6.9 Hz), δ 4.89 (d, 1H, J=2.1 Hz), δ 5.09 (dd, 1H, J=2.6, 13.1 Hz), δ 5.22 (t, 1H, J=7.3 Hz), δ 5.23 (m, 1H), δ 5.64 (dd, 1H, J=1.4, 7.5 Hz), δ 6.61 (s, 1H), δ 6.76 (s, 1H), δ 6.77 (s, 1H).

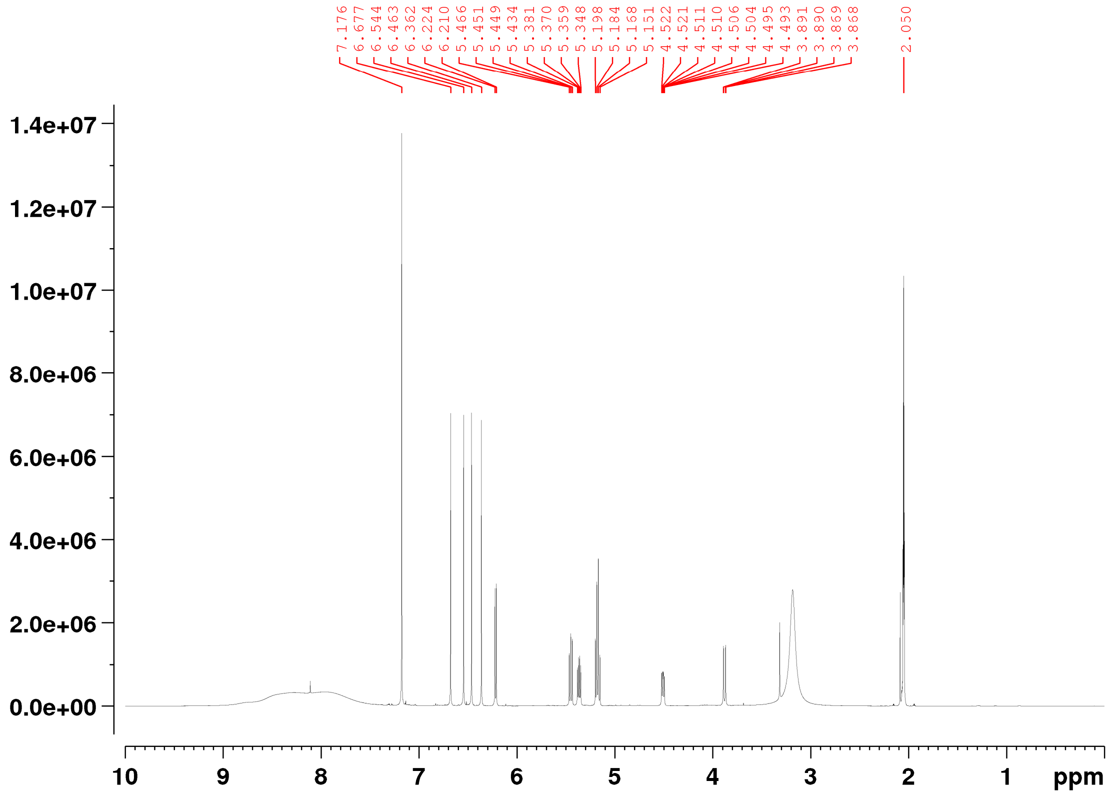

Figure S14. ^{1}H-NMR spectrum and peak assignations of casuarictin (9). ^{1}H NMR (600 MHz, acetone-*d6*) δ 3.88 (dd, 1H, J=0.9, 13.3 Hz), δ 4.51 (ddd, 1H, J=0.9, 6.7, 9.9 Hz), δ 5.17 (t, 1H, J=9.8 Hz), δ 5.18 (t, 1H, J=8.9 Hz), δ 5.36 (dd, 1H, J=6.7, 13.3 Hz), δ 5.45 (dd, 1H, J=9.2, 10.1 Hz), δ 6.21 (d, 1H, J=8.6 Hz), δ 6.36 (s, 1H), δ 6.46 (s, 1H), δ 6.54 (s, 1H), δ 6.68 (s, 1H), δ 7.18 (s, 2H).

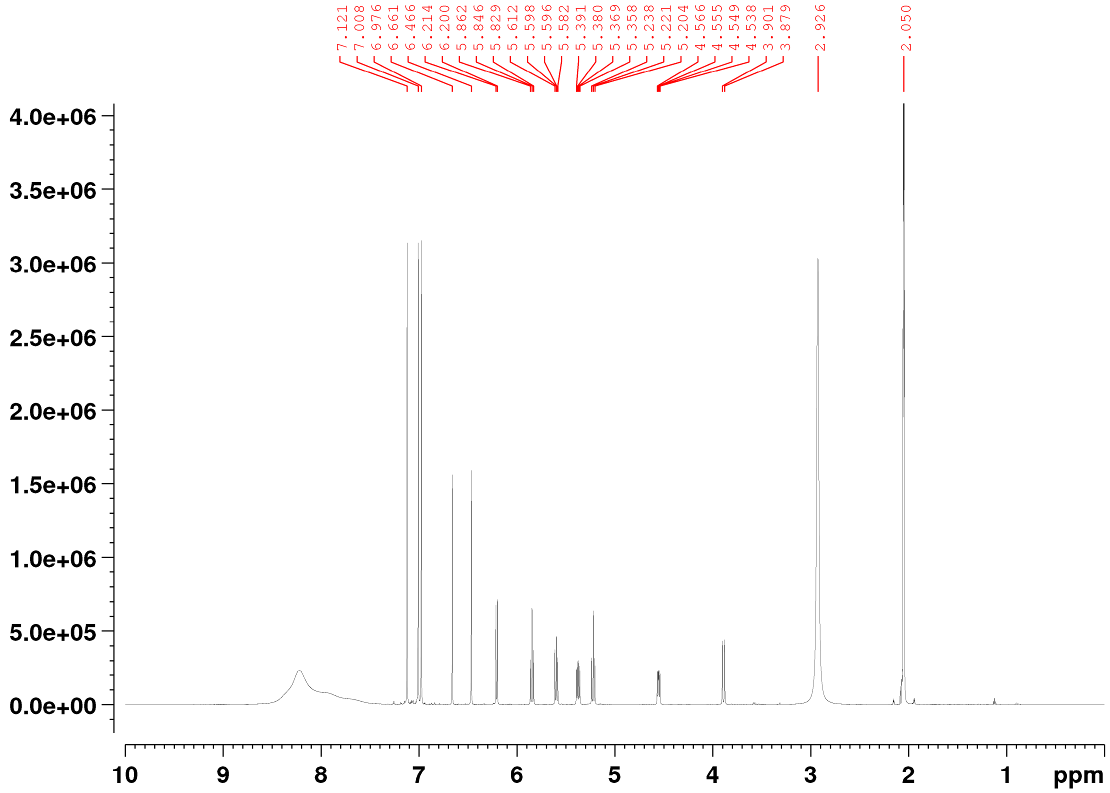

Figure S15. ^{1}H-NMR spectrum and peak assignations of tellimagrandin II (10). ^{1}H NMR (600 MHz, acetone-*d6*) δ 3.89 (d, 1H, J=13.3 Hz), δ 4.55 (dd, 1H, J=6.4, 9.9 Hz), δ 5.22 (t, 1H, J=10.0 Hz), δ 5.37 (dd, 1H, J=6.6, 13.4 Hz), δ 5.60 (dd, 1H, J=8.5, 9.4 Hz), δ 5.85 (t, 1H, J=9.8 Hz), δ 6.21 (d, 1H, J=8.3 Hz), δ 6.47 (s, 1H), δ 6.66 (s, 1H), δ 6.98 (s, 2H), δ 7.01 (s, 2H), δ 7.12 (s, 2H).

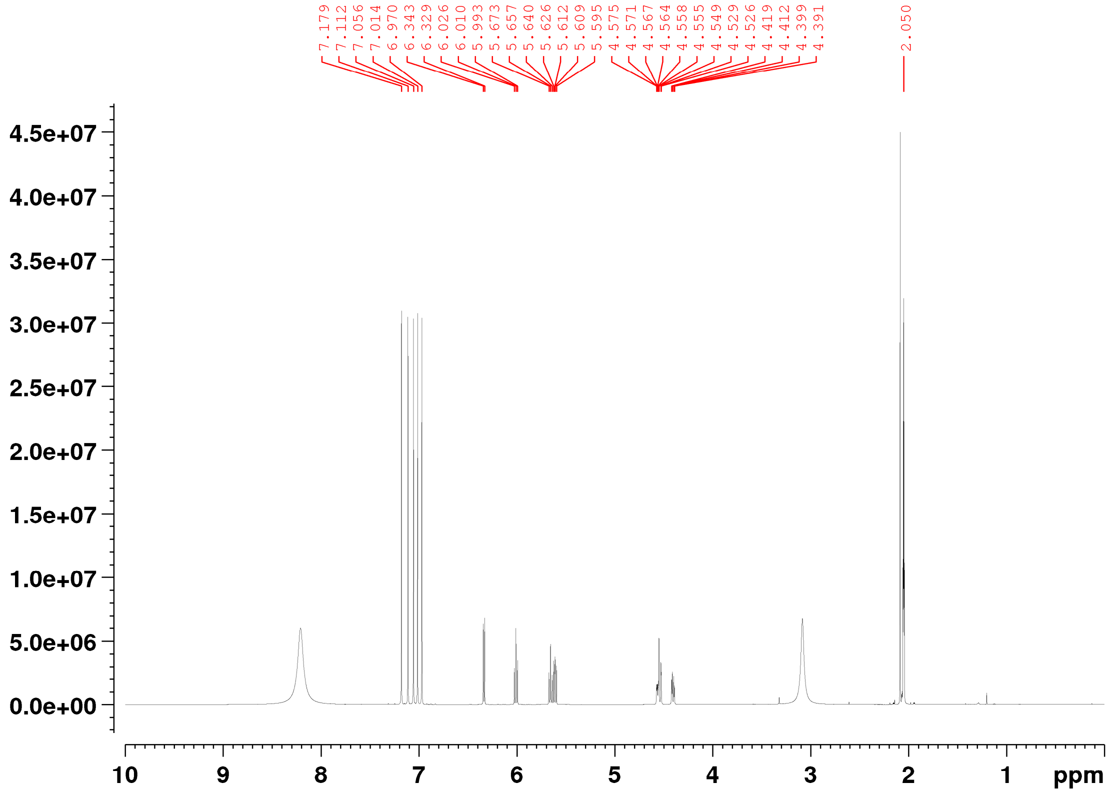

Figure S16. ^{1}H-NMR spectrum and peak assignations of 1,2,3,4,6-penta-*O*-galloyl-β-D-glucose (11). ^{1}H NMR (600 MHz, acetone-*d6*) δ 4.41 (dd, 1H, J=4.5, 12.5 Hz), δ 4.54 (m, 1H), δ 4.56 (ddd, 1H, J=1.6, 4.4, 9.9 Hz), δ 5.61 (dd, 1H, J=8.3, 9.9 Hz), δ 5.66 (t, 1H, J=9.7 Hz), δ 6.01 (t, 1H, J=9.8 Hz), δ 6.34 (d, 1H, J=8.3 Hz), δ 6.97 (s, 2H), δ 7.01 (s, 2H), δ 7.06 (s, 2H), δ 7.11 (s, 2H), δ 7.18 (s, 2H).

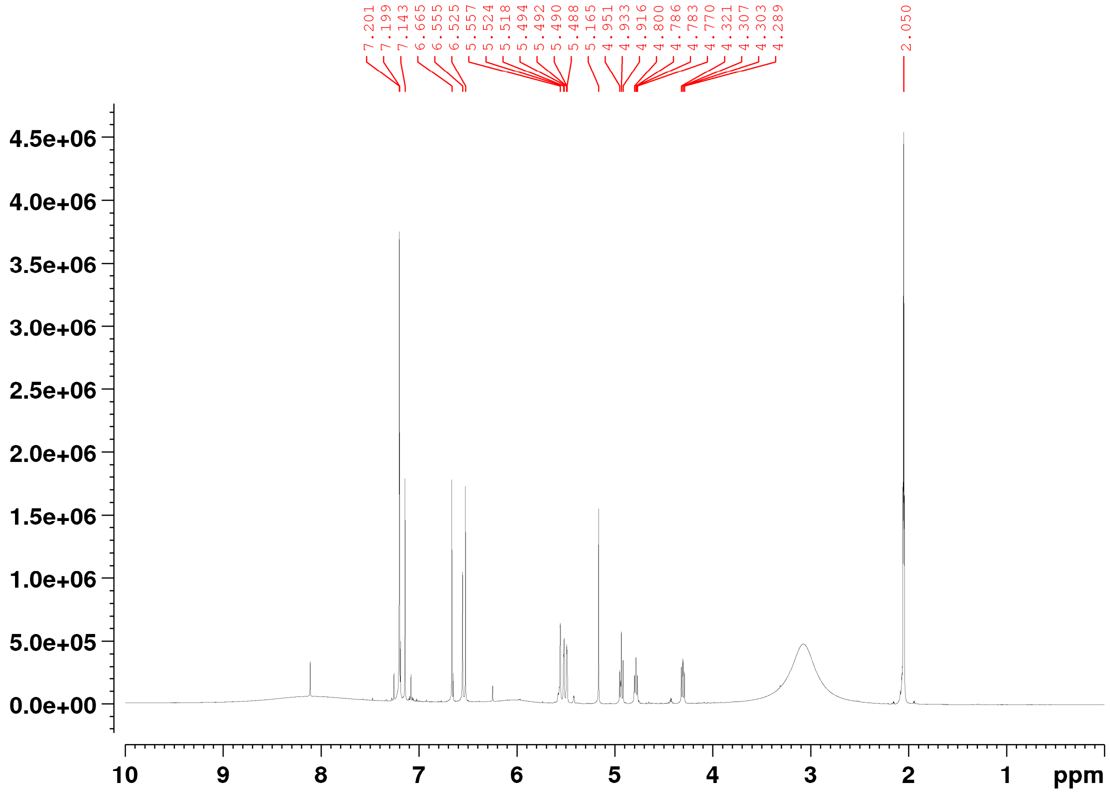

Figure S17. ^{1}H-NMR spectrum and peak assignations of geraniin (12). ^{1}H NMR (600 MHz, acetone-*d6*) δ 4.31 (dd, 1H, J=8.2, 10.8 Hz), δ 4.78 (dd, 1H, J=8.4, 9.9 Hz), δ 4.93 (t, 1H, J=10.6 Hz), δ 5.49 (br s, 1H), δ 5.52 (br s, 1H), δ 5.56 (br s, 1H), δ 6.53 (s, 1H), δ 6.56 (br s, 1H), δ 6.66 (br s, 1H), δ 7.14 (br s, 1H), δ 7.199 (br s, 1H), δ 7.201 (br s, 2H).

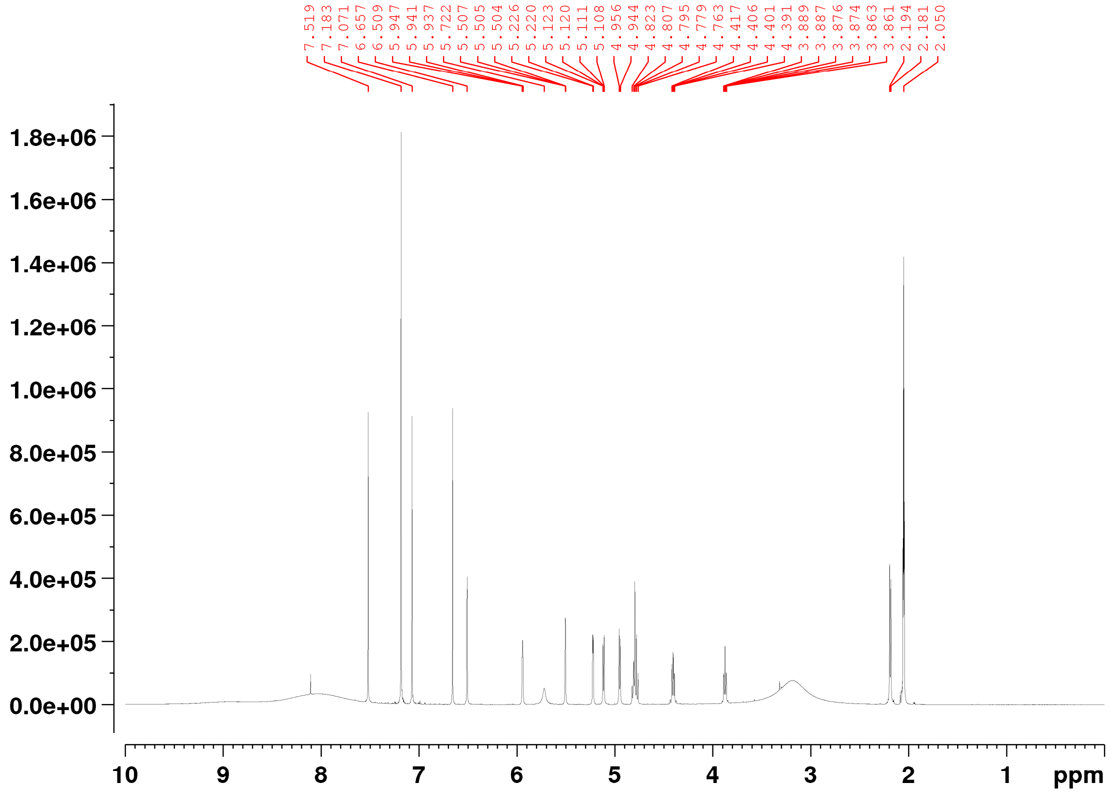

Figure S18. ^{1}H-NMR spectrum and peak assignations of chebulagic acid (13). ^{1}H NMR (600 MHz, acetone-*d6*) δ 2.19 (d, 2H, J=7.8 Hz), δ 3.87 (td, 1H, J=1.4, 11.5 Hz), δ 4.40 (dd, 1H, J=6.0, 8.8 Hz), δ 4.80 (m, 2H), δ 4.95 (d, 1H, J=7.3 Hz), δ 5.12 (dd, 1H, J=1.5, 7.2 Hz), δ 5.22 (br d, 1H, J=3.7 Hz), δ 5.51 (br s, 1H), δ 5.94 (br s, 1H), δ 6.51 (br s, 1H), δ 6.66 (s, 1H), δ 7.07 (s, 1H), δ 7.18 (s, 2H), δ 7.52 (s, 1H).

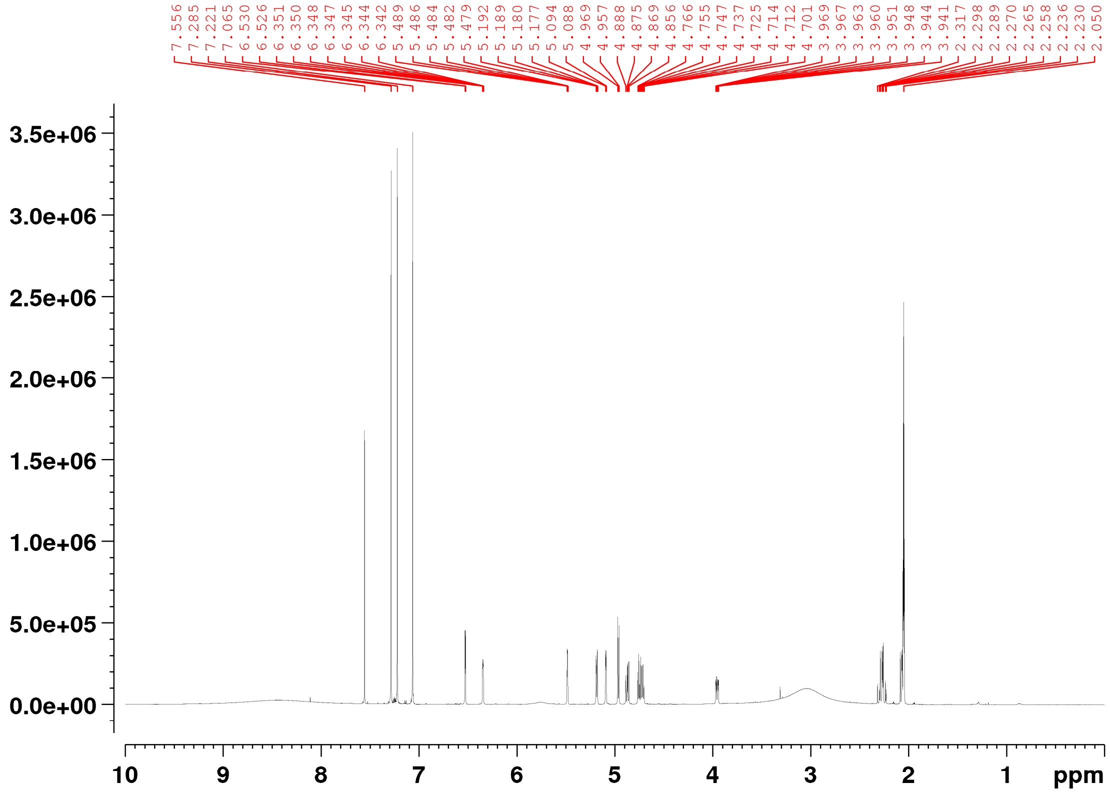

Figure S19. ¹H-NMR spectrum and peak assignations of chebulinic acid (14). ¹H NMR (600 MHz, acetone-*d6*) δ 2.27 (m, 2H), δ 3.96 (ddd, 1H, J=1.5, 4.0, 11.2 Hz), δ 4.73 (m, 2H), δ 4.87 (dd, 1H, J=7.7, 11.1 Hz), δ 4.96 (d, 1H, J=7.1 Hz), δ 5.09 (br d, 1H, J=3.5 Hz), δ 5.18 (dd, 1H, J=1.6, 7.1 Hz), δ 5.48 (br s, 1H), δ 6.35 (br s, 1H), δ 6.53 (d, 1H, J=2.3 Hz), δ 7.06 (s, 2H), δ 7.22 (s, 2H), δ 7.29 (s, 2H), δ 7.56 (s, 1H).

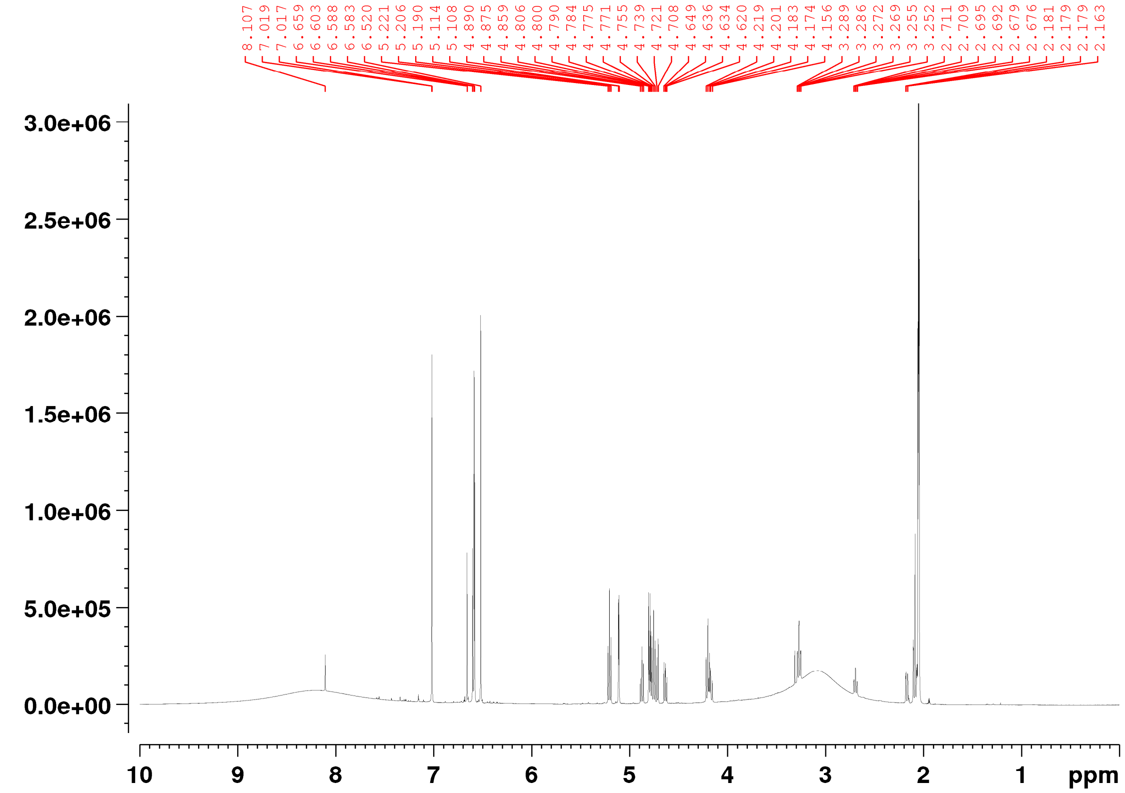

Figure S20. ^{1}H-NMR spectrum and peak assignations of punicalagin (15). Two epimers due to anomeric glucose C1 (α: β, 1.0:0.5): ^{1}H NMR (600 MHz, acetone-*d6*) δ 2.09 (dd, 1H, J=1.6, 11.2 Hz, α), δ 2.17 (dd, 1H, J=1.6, 11.4 Hz, β), δ 2.68 (td, 1H, J=1.5, 9.9 Hz, β), δ 3.27 (td, 1H, J=1.5, 10.2 Hz, α), δ 4.19 (m, 2H, both), δ 4.63 (dd, 1H, J=8.1, 9.5 Hz, β), δ 4.71 (d, 1H, J=8.0 Hz, β), δ 4.77 (m, 3H, both), δ 4.87 (t, 1H, J=9.4 Hz, β), δ 5.11 (d, 1H, J=3.4 Hz, α), δ 5.21 (t, 1H, J=9.5 Hz, α), δ 6.52 (s, 2H, both), δ 6.58 (s, 1H, α), δ 6.59 (s, 1H, α), δ 6.60 (s, 1H, β), δ 6.66 (s, 1H, β), δ 7.017 (s, 1H, α), δ 7.019 (s, 1H, β).

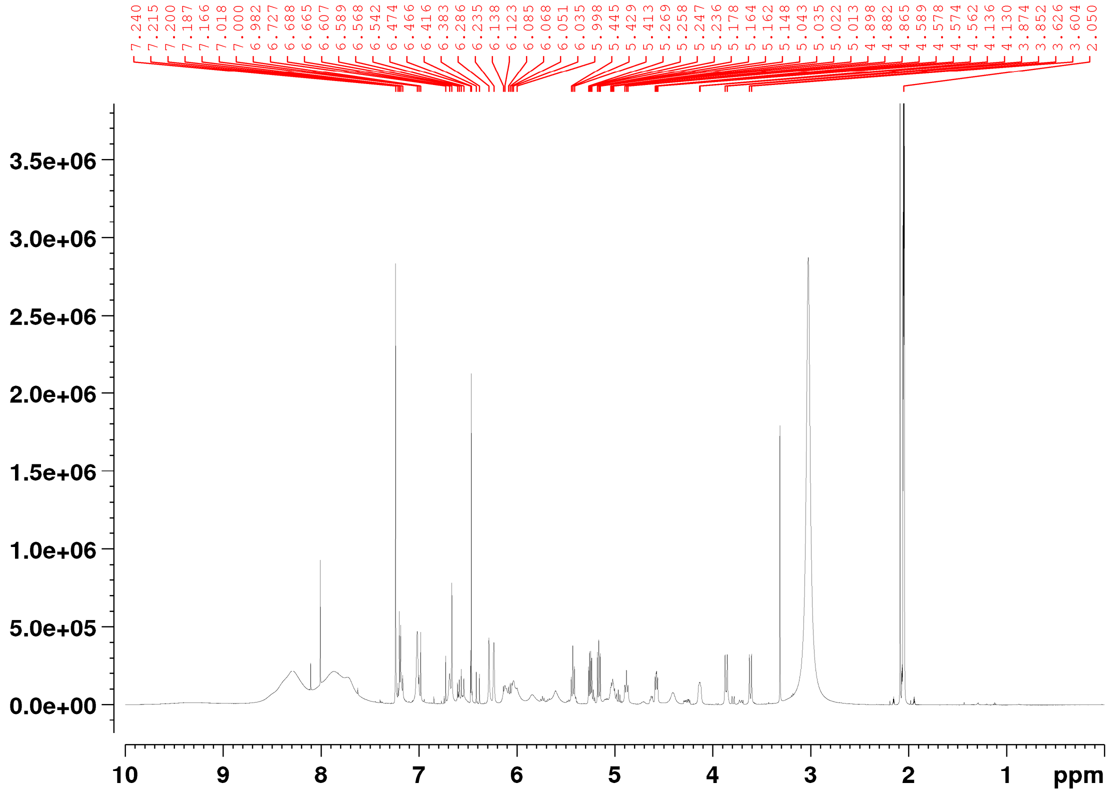

Figure S21. ^{1}H-NMR spectrum and peak assignations of oenothein B (16). Macrocyclic structure slows the interconversion of conformations broadening signals and additionally both glucose cores are anomeric increasing spectral complexity. ^{1}H NMR (acetone-*d6*) δ 3.60–6.25 (glucose proton signals of both monomeric units and the different α/β conformers), δ 6.25–7.30 (valoneyl and galloyl proton signals of both monomeric units and the different α/β conformers).

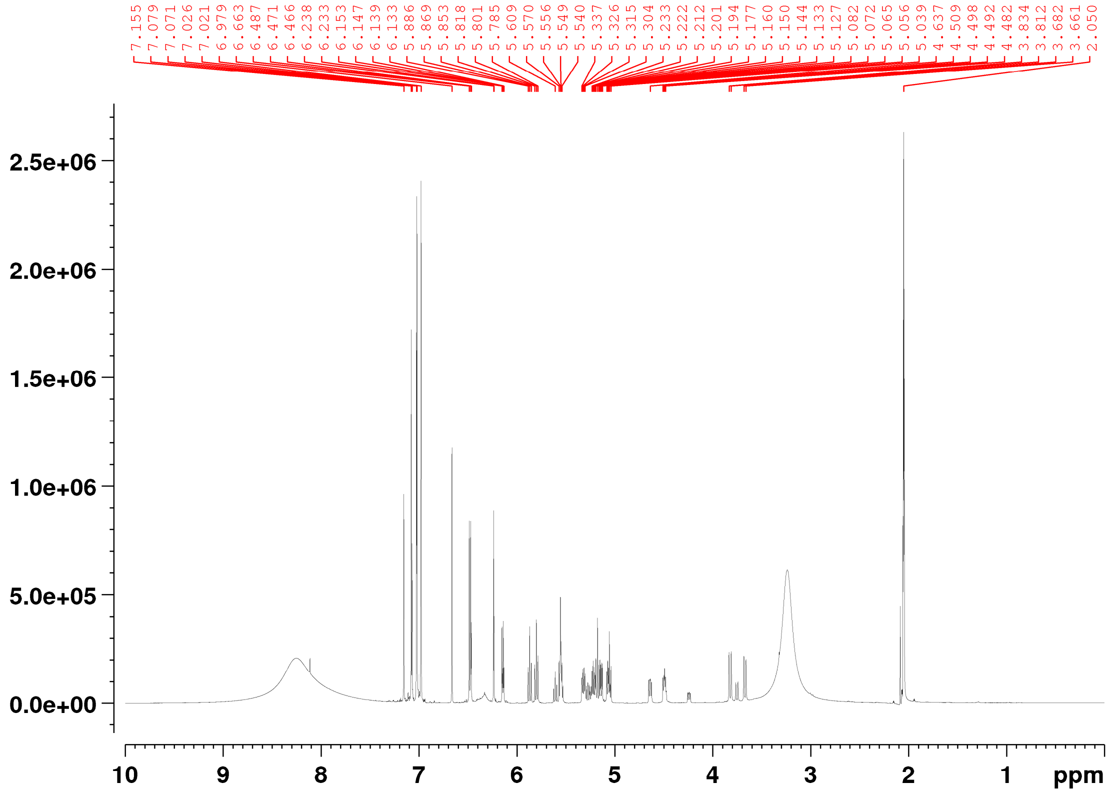

Figure S22. ^{1}H-NMR spectrum and peak assignations of rugosin E (17). Monomers designated left (L) and right (R) and α/β anomerism for the right monomer : ^{1}H NMR (600 MHz, acetone-*d6*) δ 3.67 (d, 1H, J=12.5 Hz, Rα), δ 3.75 (d, 1H, J=13.0 Hz, Rβ), δ 3.82 (d, 1H, J=13.1 Hz, L), δ 4.24 (dd, 1H, J=6.5, 10.0 Hz, Rβ), δ 4.489 (dd, 1H, J=6.1, 10.2 Hz, Lβ), δ 4.495 (dd, 1H, J=6.3, 10.1 Hz, Lα), δ 4.64 (dd, 1H, J=7.2, 9.6 Hz, Rα), δ 5.056 (t, 1H, J=10.1 Hz, Rα or Rβ), δ 5.065 (t, 1H, J=10.1 Hz, Rα or Rβ), δ 5.08 (d, 1H, J=8.1 Hz, Rβ), δ 5.14 (dd, 1H, J=3.7, 10.1 Hz, Rα), δ 5.18 (t, 1H, J=10.0 Hz, L), δ 5.22 (dd, 1H, J=6.6, 12.8 Hz, Rα), δ 5.24 (dd, 1H, J=6.5, 12.5 Hz, Rβ), δ 5.27 (dd, 1H, J=8.0, 9.5 Hz, Rβ), δ 5.318 (dd, 1H, J=6.5, 12.2 Hz, Lβ), δ 5.321 (dd, 1H, J=6.6, 13.4 Hz, Lα), δ 5.545 (dd, 1H, J=7.8, 9.7 Hz, Lα), δ 5.552 (d, 1H, J=3.7 Hz, Rα), δ 5.555 (dd, 1H, J=8.0, 9.9 Hz, Lβ), δ 5.61 (t, 1H, J=9.8, Rβ), δ 5.80 (t, 1H, J=9.8, L), δ 5.87 (t, 1H, J=10.0, Rα), δ 6.140 (d, 1H, J=8.3, Lβ), δ 6.146 (d, 1H, J=8.3, Lα), δ 6.23 (s, 1H, β), δ 6.24 (s, 1H, α), δ 6.466 (s, 1H, β), δ 6.471 (s, 2H, α/β), δ 6.49 (s, 1H, α), δ 6.66 (s, 2H, α/β), δ 6.98 (s, 4H, α/β), δ 7.02 (s, 4H, α/β), δ 7.03 (s, 4H, α/β), δ 7.07 (s, 2H, β), δ 7.08 (s, 2H, α), δ 7.16 (s, 2H, α/β).

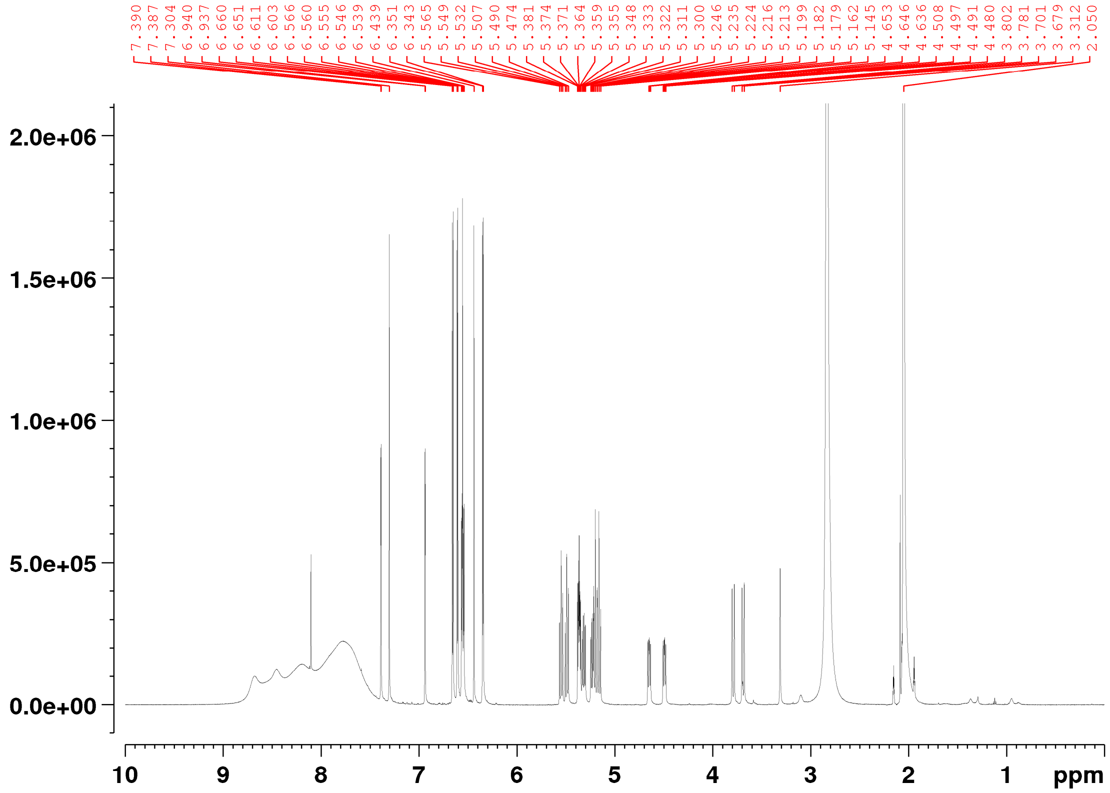

Figure S23. ^{1}H-NMR spectrum and peak assignations of agimoniin (18). ^{1}H NMR (600 MHz, acetone-*d6*) δ 3.69 (d, 1H, J=13.0 Hz), δ 3.79 (d, 1H, J=12.8 Hz), δ 4.49 (dd, 1H, J=6.5, 10.2 Hz), δ 4.65 (dd, 1H, J=6.5, 10.2 Hz), δ 5.16 (t, 1H, J=10.4 Hz), δ 5.20 (t, 1H, J=10.3 Hz), δ 5.23 (dd, 1H, J=6.5, 13.2 Hz), δ 5.32 (dd, 1H, J=6.6, 13.4 Hz), δ 5.36 (dd, 1H, J=3.9, 9.5 Hz), δ 5.37 (dd, 1H, J=3.8, 9.6 Hz), δ 5.49 (t, 1H, J=9.8 Hz), δ 5.55 (t, 1H, J=9.8 Hz), δ 6.34 (s, 1H), δ 6.35 (s, 1H), δ 6.44 (s, 1H), δ 6.54 (d, 1H, J=4.0 Hz), δ 6.55 (s, 1H), δ 6.56 (d, 1H, J=4.0 Hz), δ 6.60 (s, 1H), δ 6.61 (s, 1H), δ 6.65 (s, 1H), δ 6.66 (s, 1H), δ 6.94 (d, 1H, J=1.9 Hz), δ 7.30 (s, 1H), δ 7.39 (d, 1H, J=1.9 Hz).

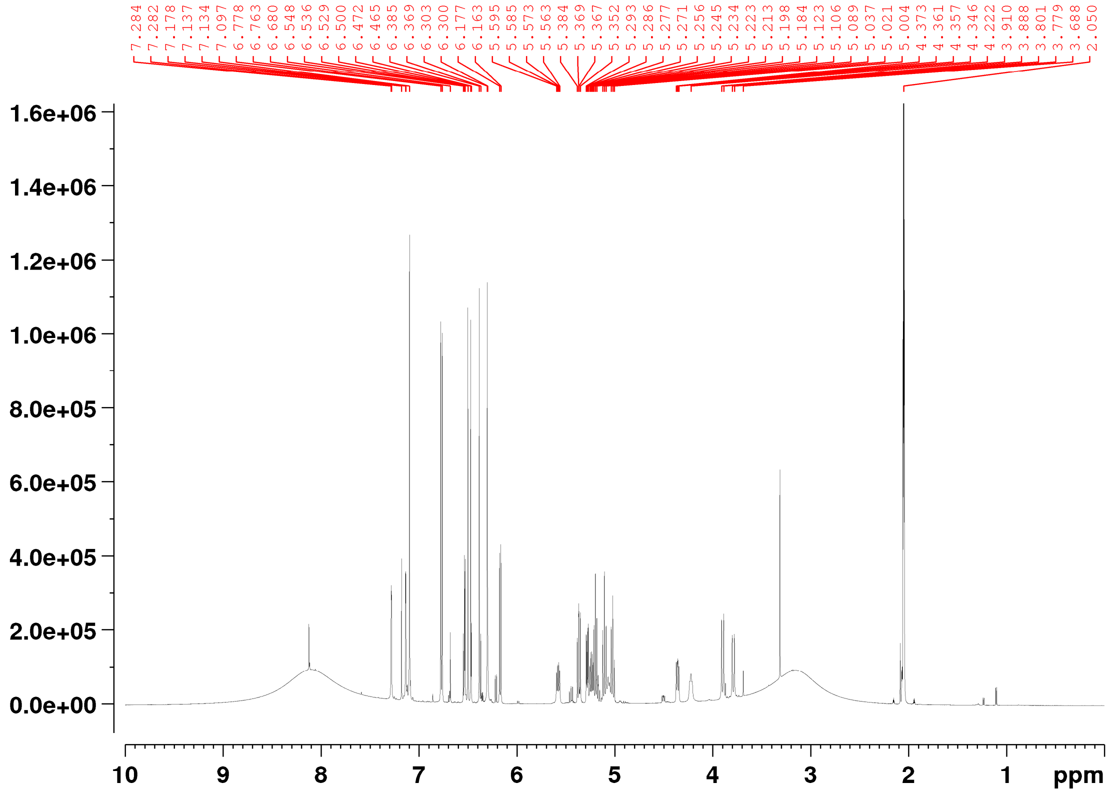

Figure S24. ^{1}H-NMR spectrum and peak assignations of sanguiin H-6 (19). ^{1}H NMR (600 MHz, acetone-*d6*) δ 3.79 (d, 1H, J=13.1 Hz), δ 3.90 (d, 1H, J=12.9 Hz), δ 4.22 (br s, 1H), δ 4.36 (dd, 1H, J=7.0, 9.4 Hz), δ 5.02 (t, 1H, J=10.2 Hz), δ 5.10 (t, 2H, J=10.1 Hz), δ 5.20 (t, 2H, J=8.8 Hz), δ 5.24 (dd, 1H, J=6.6, 13.4 Hz), δ 5.28 (dd, 1H, J=4.0, 9.4 Hz), δ 5.37 (dd, 1H, J=9.1, 10.2 Hz), δ 5.58 (dd, 1H, J=6.5, 13.2 Hz), δ 6.17 (d, 1H, J=8.5 Hz), δ 6.300 (s, 1H), δ 6.303 (s, 1H), δ 6.38 (s, 1H), δ 6.47 (s, 1H), δ 6.50 (s, 1H), δ 6.53 (d, 1H, J=4.0 Hz), δ 6.76 (s, 1H), δ 6.78 (s, 1H), δ 7.10 (s, 1H), δ 7.14 (d, 1H, J=1.9 Hz), δ 7.28 (d, 1H, J=1.7 Hz).

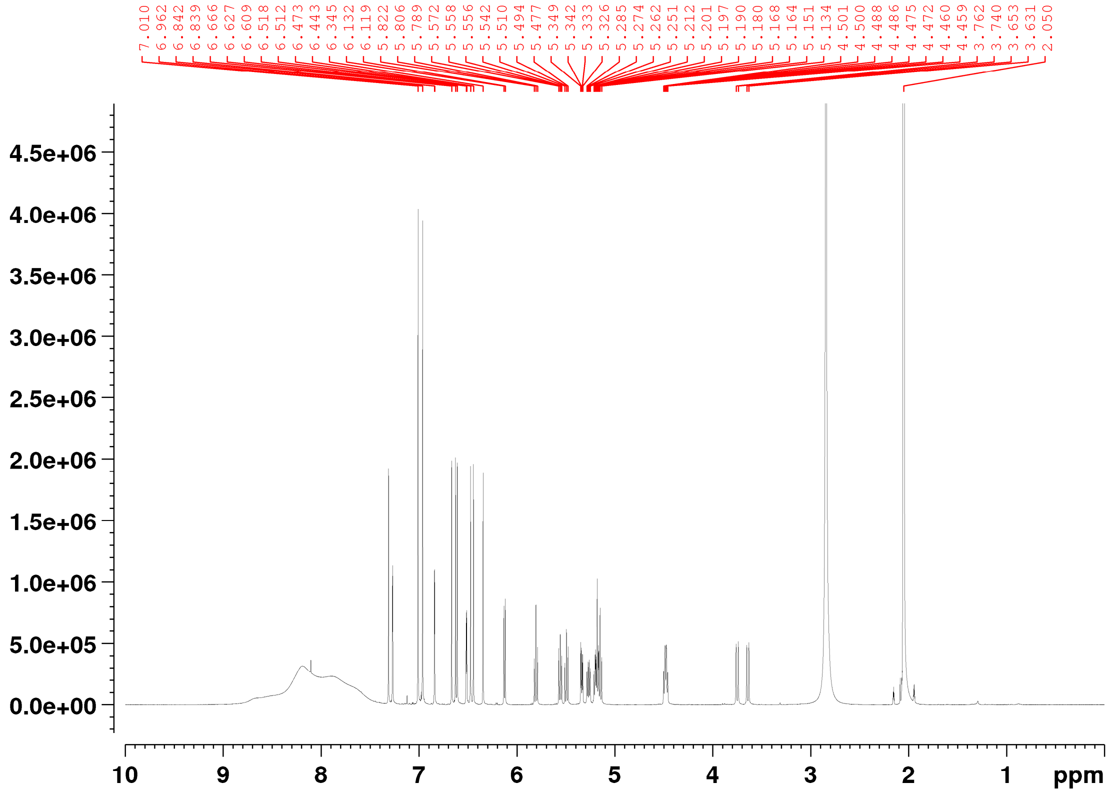

Figure S25. ^{1}H-NMR spectrum and peak assignations of gemin A (20). ^{1}H NMR (600 MHz, acetone-*d6*) δ 3.64 (d, 1H, J=13.3 Hz), δ 3.75 (d, 1H, J=13.4 Hz), δ 4.479 (dd, 1H), δ 4.481 (dd, 1H), δ 5.15 (t, 1H, J=10.3 Hz), δ 5.18 (d, 1H, J=10.0 Hz), δ 5.20 (dd, 1H, J=6.3, 12.9 Hz), δ 5.27 (d, 1H, J=6.6, 13.4 Hz), δ 5.34 (dd, 1H, J=4.1, 9.3 Hz), δ 5.49 (t, 1H, J=9.8 Hz), δ 5.56 (dd, 1H, J=8.4, 9.5 Hz), δ 5.81 (t, 1H, J=9.8 Hz), δ 6.13 (d, 1H, J=8.3 Hz), δ 6.35 (s, 1H), δ 6.44 (s, 1H), δ 6.47 (s, 1H), δ 6.51 (d, 1H, J=4.0 Hz), δ 6.61 (s, 1H), δ 6.63 (s, 1H), δ 6.67 (s, 1H), δ 6.84 (d, 1H, J=1.9 Hz), δ 6.96 (s, 2H), δ 7.01 (s, 2H), δ 7.27 (d, 1H, J=1.9 Hz), δ 7.31 (s, 1H).

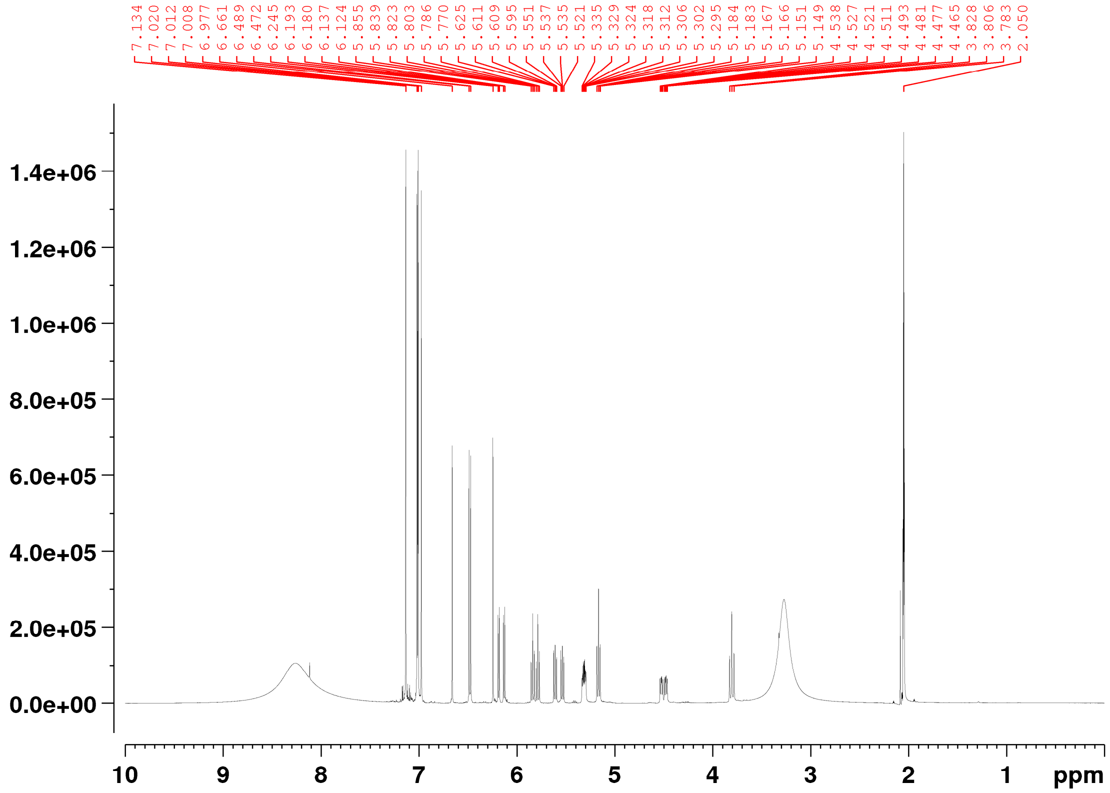

Figure S26. ^{1}H-NMR spectrum and peak assignations of rugosin D (21). ^{1}H NMR (600 MHz, acetone-*d6*) δ 3.79 (d, 1H, J=13.4 Hz), δ 3.82 (d, 1H, J=13.5 Hz), δ 4.48 (dd, 1H, J=7.2, 9.9 Hz), δ 4.52 (dd, 1H, J=6.3, 10.0 Hz), δ 5.166 (t, 1H, J=10.0 Hz), δ 5.167 (t, 1H, J=10.0 Hz), δ 5.312 (dd, 1H, J=6.5, 13.4 Hz), δ 5.318 (dd, 1H, J=6.6, 13.4 Hz), δ 5.36 (dd, 1H, J=8.3, 9.4 Hz), δ 5.61 (dd, 1H, J=8.4, 9.5 Hz), δ 5.79 (t, 1H, J=9.8 Hz), δ 5.84 (t, 1H, J=9.8 Hz), δ 6.13 (d, 1H, J=8.3 Hz), δ 6.19 (d, 1H, J=8.3 Hz), δ 6.24 (s, 1H), δ 6.47 (s, 1H), δ 6.49 (s, 1H), δ 6.66 (s, 1H), δ 6.98 (s, 2H), δ 7.008 (s, 2H), δ 7.012 (s, 2H), δ 7.02 (s, 2H), δ 7.134 (s, 2H), δ 7.136 (s, 1H).

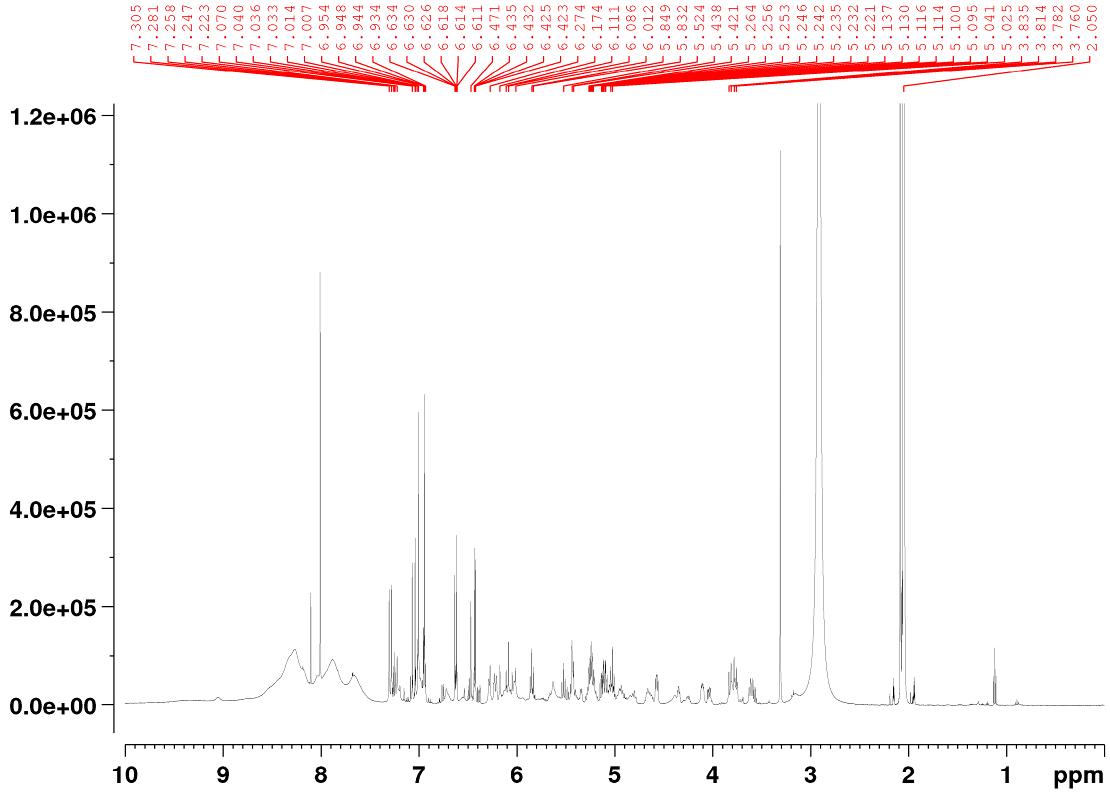

Figure S27. ^{1}H-NMR spectrum and peak assignations of oenothein A (22). Macrocyclic structure slows the interconversion of conformations broadening signals and additionally all glucose cores are anomeric increasing spectral complexity. ^{1}H NMR (600 MHz, acetone-*d6*) δ 3.50–6.32 (glucose proton signals of all monomeric units and the different α/β conformers), δ 6.35–7.32 (valoneyl and galloyl proton signals of all monomeric units and the different α/β conformers).

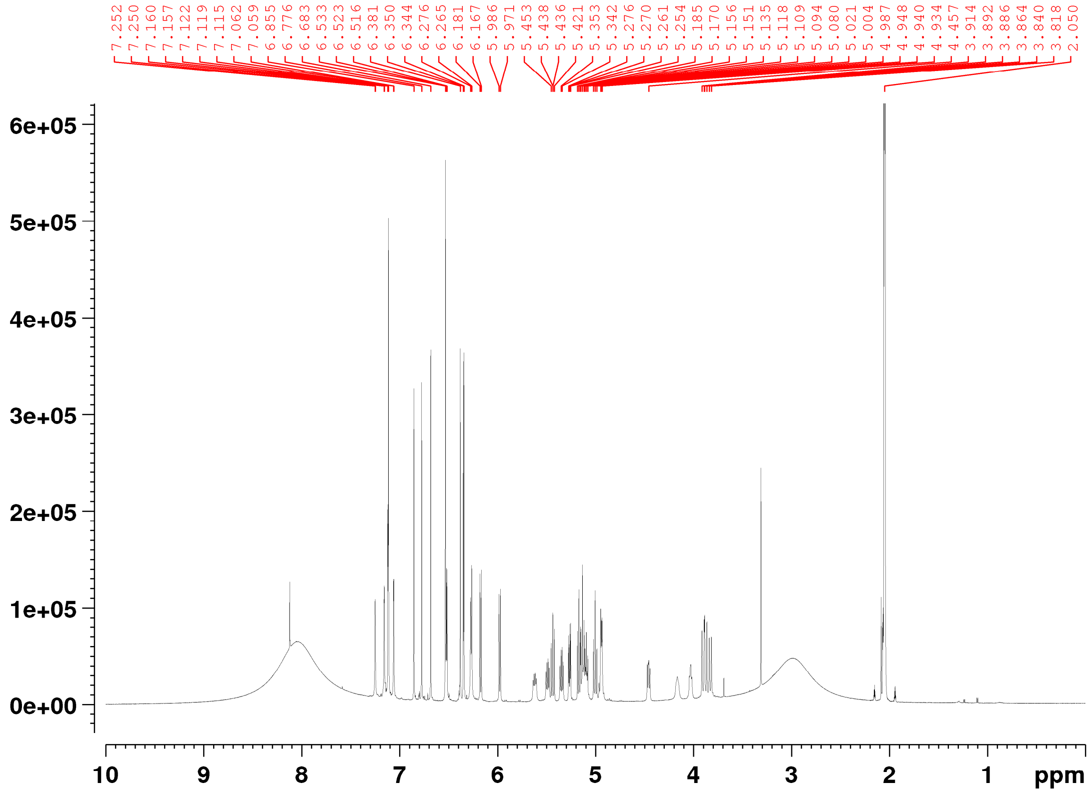

Figure S28. ^{1}H-NMR spectrum and peak assignations of lambertianin C (23). ^{1}H NMR (600 MHz, acetone-*d6*) δ 3.83 (d, 1H, J=13.2 Hz), δ 3.88 (d, 1H, J=13.3 Hz), δ 3.90 (d, 1H, J=13.3 Hz), δ 4.03 (dd, 1H, J=6.1, 8.6), δ 4.17 (br s, 1H), δ 4.46 (ddd, 1H, J=1.0, 6.8, 9.8 Hz), δ 4.94 (t, 2H), δ 5.00 (t, 1H, J=10.2 Hz), δ 5.09 (t, 1H, J=8.8 Hz), δ 5.11 (dd, 1H, J=8.4, 12.8 Hz), δ 5.13 (t, 1H, J=10.1 Hz), δ 5.17 (t, 1H, J=8.9 Hz), δ 5.27 (dd, 1H, J=4.0, 9.4 Hz), δ 5.35 (dd, 1H, J=6.7, 13.3 Hz), δ 5.44 (dd, 1H, J=9.3, 10.1 Hz), δ 5.49 (dd, 1H, J=6.5, 13.2 Hz), δ 5.62 (dd, 1H, J=6.4, 13.4 Hz), δ 5.98 (d, 1H, J=8.5 Hz), δ 6.17 (d, 1H, J=8.5 Hz), δ 6.26 (s, 1H), δ 6.28 (s, 1H), δ 6.34 (s, 1H), δ 6.35 (s, 1H), δ 6.38 (s, 1H), δ 6.52 (d, 1H, J=3.9 Hz), δ 6.53 (s, 2H), δ 6.68 (s, 1H), δ 6.78 (s, 1H), δ 6.85 (s, 1H), δ 7.06 (d, 1H, J=1.8 Hz), δ 7.1 (s, 2H), δ 7.12 (d, 1H, J=2.0 Hz), δ 7.16 (d, 1H, J=1.7 Hz), δ 7.25 (d, 1H, J=1.5 Hz).

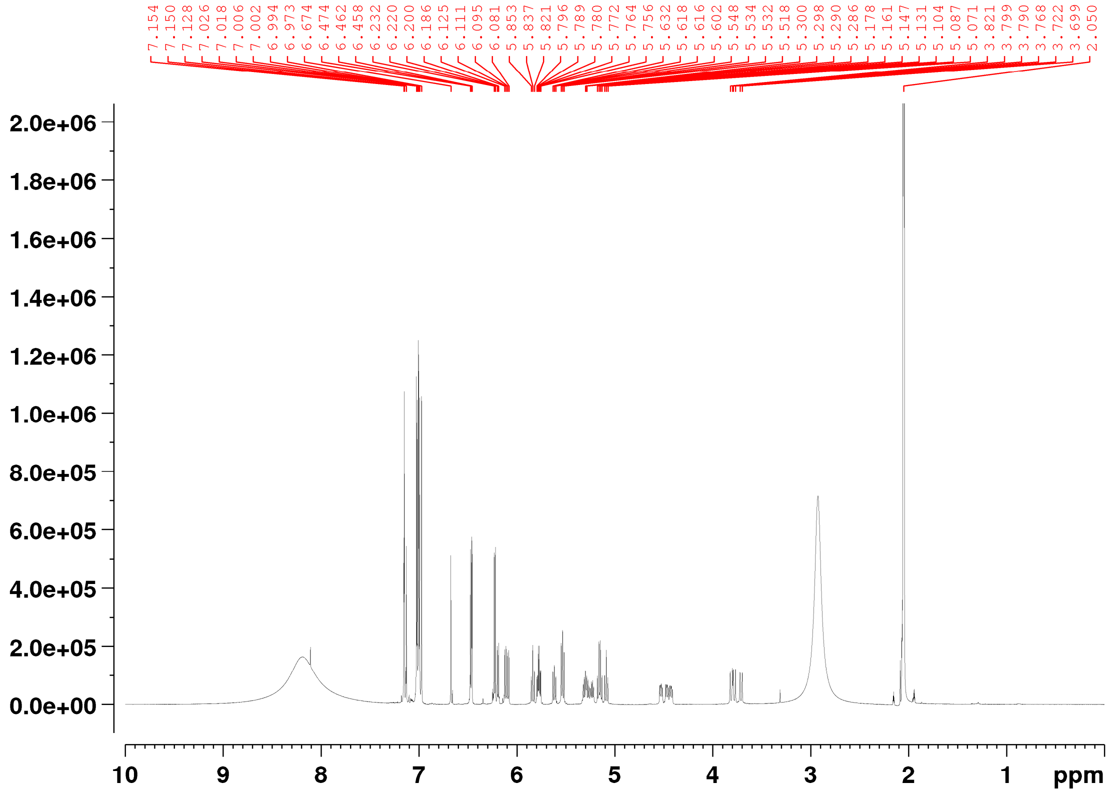

Figure S29. ^{1}H-NMR spectrum and peak assignations of rugosin G (24). ^{1}H NMR (600 MHz, acetone-*d6*) δ 3.71 (d, 1H, J=13.3 Hz), δ 3.78 (d, 1H, J=13.3 Hz), δ 3.81 (d, 1H, J=13.3 Hz), δ 4.26 (dd, 1H, J=6.8, 10.0 Hz), δ 4.47 (dd, 1H, J=7.0, 9.7 Hz), δ 4.5 (dd, 1H, J=6.6, 10.1 Hz), δ 5.09 (t, 1H, J=10.0 Hz), δ 5.15 (t, 1H, J=10.0 Hz), δ 5.16 (t, 1H, J=10.0 Hz), δ 5.23 (dd, 1H, J=6.6, 13.5 Hz), δ 5.28 (dd, 1H, J=6.6, 13.7 Hz), δ 5.31 (dd, 1H, J=6.5, 13.4 Hz), δ 5.53 (dd, 2H, J=8.5, 9.5 Hz), δ 5.62 (dd, 1H, J=8.4, 9.5 Hz), δ 5.77 (t, 1H, J=9.8 Hz), δ 5.78 (t, 1H, J=9.8 Hz), δ 5.84 (t, 1H, J=9.8 Hz), δ 6.09 (d, 1H, J=8.3 Hz), δ 6.12 (d, 1H, J=8.3 Hz), δ 6.19 (d, 1H, J=8.3 Hz), δ 6.22 (s, 1H), δ 6.23 (s, 1H), δ 6.458 (s, 1H), δ 6.462 (s, 1H), δ 6.47 (s, 1H), δ 6.67 (s, 1H), δ 6.97 (s, 2H), δ 6.99 (s, 2H), δ 7.002 (s, 2H), δ 7.006 (s, 2H), δ 7.02 (s, 2H), δ 7.03 (s, 2H), δ 7.13 (s, 1H), δ 7.150 (s, 2H), δ 7.153 (s, 1H).
